# Circulating extracellular vesicles release oncogenic miR-424 in experimental models and patients with aggressive prostate cancer

**DOI:** 10.1038/s42003-020-01642-5

**Published:** 2021-01-26

**Authors:** Domenico Albino, Martina Falcione, Valeria Uboldi, Dada Oluwaseyi Temilola, Giada Sandrini, Jessica Merulla, Gianluca Civenni, Aleksandra Kokanovic, Alessandra Stürchler, Dheeraj Shinde, Mariangela Garofalo, Ricardo Pereira Mestre, Vera Constâncio, Martha Wium, Jacopo Burrello, Nicolò Baranzini, Annalisa Grimaldi, Jean-Philippe Theurillat, Daniela Bossi, Lucio Barile, Rui M. Henrique, Carmen Jeronimo, Luiz Fernando Zerbini, Carlo V. Catapano, Giuseppina M. Carbone

**Affiliations:** 1 Institute of Oncology Research (IOR), Università della Svizzera Italiana (USI), 6500 Bellinzona, Switzerland; 2 International Centre for Genetic Engineering and Biotechnology (ICGEB), Cape Town, South Africa and Integrative Biomedical Sciences Division, Faculty of Health Sciences, University of Cape Town, Cape Town, South Africa; 3 Department of Pharmaceutical and Pharmacological Sciences, University of Padua, Padova, Italy; 4 Medical Oncology, Oncology Institute of Southern Switzerland, 6500 Bellinzona, Switzerland; 5 Cancer Biology and Epigenetics Group, Research Center (CI-IPOP), Portuguese Oncology Institute of Porto (IPO Porto), Porto, Portugal; 6 Laboratory for Cardiovascular Theranostics, Cardiocentro Ticino Foundation, USI, Lugano, Switzerland; 7 Department of Biotechnology and Life Science, Università degli Studi dell’Insubria, Varese, Italy; 8 Department of Pathology, Portuguese Oncology Institute of Porto, Porto, Portugal; 9 Department of Pathology and Molecular Immunology, Institute of Biomedical Sciences Abel Salazar-University of Porto (ICBAS-UP), Porto, Portugal

**Keywords:** Cancer, Prostate cancer

## Abstract

Extracellular vesicles (EVs) are relevant means for transferring signals across cells and facilitate propagation of oncogenic stimuli promoting disease evolution and metastatic spread in cancer patients. Here, we investigated the release of miR-424 in circulating small EVs or exosomes from prostate cancer patients and assessed the functional implications in multiple experimental models. We found higher frequency of circulating miR-424 positive EVs in patients with metastatic prostate cancer compared to patients with primary tumors and BPH. Release of miR-424 in small EVs was enhanced in cell lines (LNCaP^abl^), transgenic mice (Pb-Cre4;Pten^flox/flox^;Rosa26^ERG/ERG^) and patient-derived xenograft (PDX) models of aggressive disease. EVs containing miR-424 promoted stem-like traits and tumor-initiating properties in normal prostate epithelial cells while enhanced tumorigenesis in transformed prostate epithelial cells. Intravenous administration of miR-424 positive EVs to mice, mimicking blood circulation, promoted miR-424 transfer and tumor growth in xenograft models. Circulating miR-424 positive EVs from patients with aggressive primary and metastatic tumors induced stem-like features when supplemented to prostate epithelial cells. This study establishes that EVs-mediated transfer of miR-424 across heterogeneous cell populations is an important mechanism of tumor self-sustenance, disease recurrence and progression. These findings might indicate novel approaches for the management and therapy of prostate cancer.

## Abstract (web summary)

Following on reports that miR-424 expression promotes oncogenesis, Domenico Albino et al. find that extracellular vesicles (EVs) in the plasma of prostate cancer patients secrete miR-424. Using cell-based and animal models, they demonstrate that EV-mediated release of miR-424 can transfer oncogenic signals across cells to promote recurrence and metastatic progression.

## Introduction

Prostate cancer is the most frequent cause of cancer-related mortality in men^1^. Aggressive forms of prostate cancers rapidly progress to metastatic, hormone-refractory, and lethal disease. Paracrine propagation of oncogenic signals by horizontal transfer across cell types is a dynamic process that can promote cell plasticity, metastatic spread and treatment resistance^2–4^. However, the underlying mechanisms are still elusive. In this context, small extracellular vesicles (EVs) or exosomes of 30–150 nm in diameter can serve as efficient means for transferring intracellular content, allowing cell-to-cell communication within the tumor microenvironment and at distant sites^2,3^. Small EVs contain nucleic acids, lipids, and proteins^2^. MicroRNAs (miRNAs) are frequently packaged into exosomes and are secreted in the extracellular environment^5–7^. Exosome-encapsulated miRNAs are extremely stable and are found in all body fluids, including blood^6–9^. Therefore, miRNAs loaded in exosomes released by normal and cancer cells can influence the behavior of other cell types by transferring their tumor-promoting or tumor-suppressing capability^2,10^.

We have shown that the transcription factor ESE3/EHF is frequently downregulated in prostate cancer and its loss induces cell plasticity and acquisition of mesenchymal, stem-like and tumor-initiating properties^11–13^. We found that loss of ESE3/EHF led to increased expression of miR-424 that acted as a potent oncogenic effector activating a complex program involving COP1 repression and induction of multiple oncogenic transcription factors, including STAT3, c-JUN, and ETV1^14^. Interestingly, transient expression of miR-424 in immortalized normal prostate epithelial cells was sufficient to promote stem-like and tumor-initiating features, suggesting that transfer of miR-424 across distinct cell types could modify persistently the recipient cells and amplify the extent of miR-424 oncogenic signaling^14^.

In this study, we investigated whether miR-424 was secreted in small EVs or exosomes released by prostate tumors and whether it could act in paracrine and endocrine manner in order to promote tumorigenic phenotypes in low tumorigenic cells at both proximal and distal sites. We detected EVs carrying miR-424 in plasma of prostate cancer patients and examined the functional implications of EVs-released miR-424 using multiple in vitro and in vivo experimental models. We show that miR-424-loaded EVs from cell lines, mouse models, patient-derived xenografts (PDX) and patient samples promote stem-like features and tumorigenic capability in recipient cells. Therefore, EVs-mediated release of miR-424 can serve as an efficient means for transferring oncogenic signals across cells in the surrounding microenvironment and at distal metastatic sites promoting disease recurrence and progression.

## Results

### Aggressive prostate cancers release miR-424 containing EVs

**Fig. 1:**
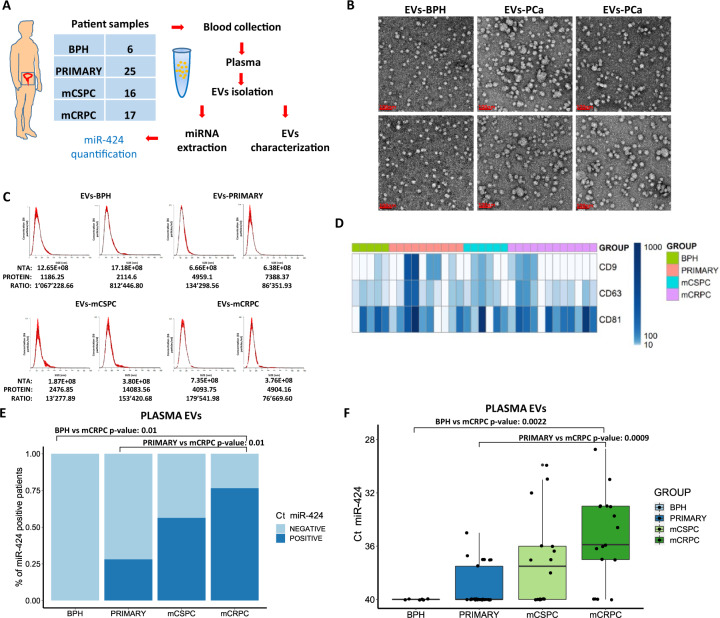
Metastatic prostate cancers release miR-424 containing extracellular vesicles in patient plasma. **A** Scheme of the experimental plan for isolation and characterization of EVs from patient plasma. **B** and **C** Representative images of transmission electron microscopy (TEM) and nanoparticle tracking analysis (NTA) of patient-derived EVs. EVs protein concentrations and ratios between NTA counts vs. protein are reported. **D** Expression of EVs surface markers in patient-derived EVs determined by MACSPlex analysis. **E** Percentage of miR-424-positive EVs isolated from plasma of patients with BPH and prostate cancer. **F** miR-424 expression levels (Ct values) in EVs from patient plasma. Comparisons with significant *p*-value (<0.05) are indicated and only significant *p*-values are reported. *n* = 6 BPH, *n* = 25 PRIMARY, *n* = 16 mCSPC, *n* = 17 mCRPC. In NTA panels the unit of measurements were particles/mL.

We hypothesized that miR-424-positive tumors could release miRNA-loaded exosomes in the extracellular environment and blood circulation. To test this, we collected blood and isolated small EVs from plasma of patients (*n* = 64) with benign prostatic hyperplasia (BPH) (*n* = 6), primary (*n* = 25), metastatic castration-sensitive (mCSPC) (*n* = 16), and metastatic castration-resistant (mCRPC) (*n* = 17) prostate cancers (Fig. 1A). Both morphology and size (<100 nm) were consistent with the isolation of small EVs as determined by transmission electron microscopy (TEM) and nanoparticle-tracking analysis (NTA) (Fig. 1B and C), in accordance with the standard MISEV guidelines^15^. MACS-PLEX analysis showed expression of the EVs-marker proteins CD9, CD63, CD81 in representative patient samples from all groups (Fig. 1D). Immunoblotting confirmed the presence of CD81 and the absence of contaminating cellular proteins such as GRP94 and calnexin (Supplementary Figs. 1 and 9). We measured the level of miR-424 in plasma EVs from BPH and tumor samples by RT-qPCR. miR-424 was not detected (Ct ≥ 40) in BPH samples, while the percentage of positivity increased from primary tumors to mCSPCs and mCRPCs (Fig. 1E). Notably, mCRPC-derived EVs had higher levels of miR-424 compared to normal and primary tumors, while the difference was not statistically significant for mCSPCs (Fig. 1F). These findings were in line with an impact of miR-424 in progression to metastatic and hormone-refractory prostate cancer. Importantly, our present finding implied that EVs-mediated release of miR-424 in the extracellular environment and blood circulation could expand the range and amplify the oncogenic effects of this miRNA in prostate cancer patients.

### miR-424-loaded EVs drive stem-like and tumorigenic traits in recipient cells

**Fig. 2:**
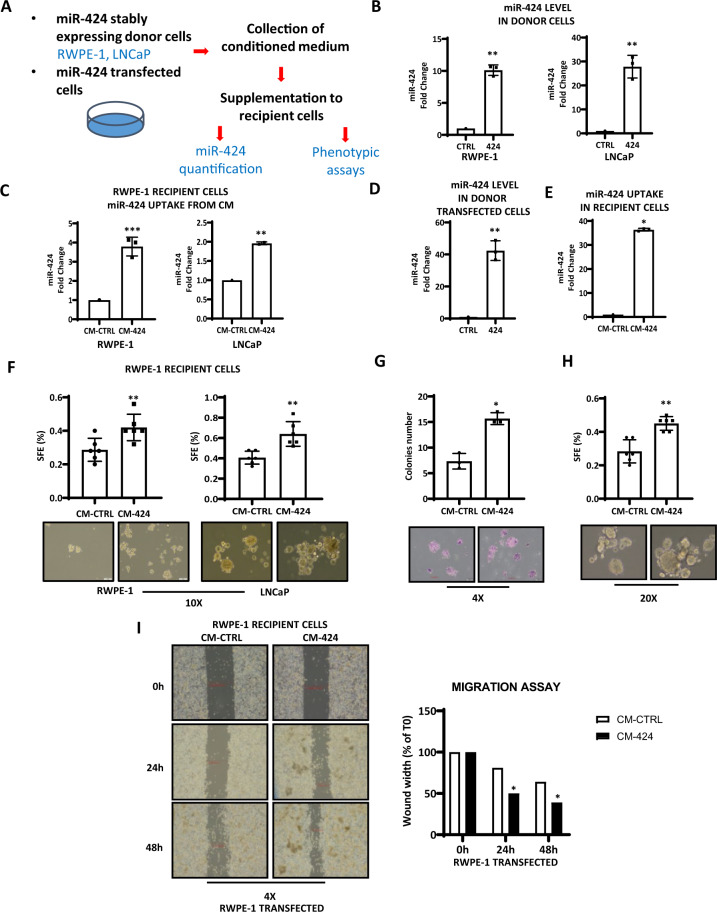
Paracrine impact of secreted miR-424. **A** Experimental design for phenotypic analysis. **B** miR-424 levels in RWPE-1 and LNCaP donor cells stably expressing miR-424 or control plasmid. **C** miR-424 levels in recipient cells (RWPE-1) and (LNCaP) following the addition of conditioned medium (CM) from donor cells with (CM-424) or without (CM-CTRL) stable expression of miR-424. **D** miR-424 level in RWPE-1 cells transfected with pre-miR-424 (424) or negative control pre-miRNA (CTRL). **E** miR-424 transfer in recipient RWPE-1 cells following incubation with CM from control or pre-miR-424 transfected RWPE-1 cells. **F** Tumor-sphere forming efficiency (SFE) of RWPE-1 recipient cells incubated with CM from RWPE-1 and LNCaP donor cells with (CM-424) or without (CM-CTRL) miR-424 expression. **G** and **H** Colony (**G**) and tumor-sphere formation (**H**) after supplementation of CM from control or pre-miR-424 transfected RWPE-1 cells. Representative images are shown at bottom of panels **F** and **G**. **I** Cell migration capacity measured in wound-healing assay of RWPE-1 cells after supplementation of CM from control and pre-miR-424 transfected RWPE-1 cells. Right: quantification of wound width closure percentage. *p ≤ 0.05, **p ≤ 0.01 by two-tailed Student’s *t*-test.

To investigate the function of secreted miR-424, we collected conditioned medium (CM) from prostate donor cells engineered to stably express miR-424 and assessed the effects in miR-424-negative recipient cells (Fig. 2A). Both RWPE-1 miR-424 and LNCaP miR-424 donor cells had significantly higher levels of miR-424 than corresponding control cells (Fig. 2B). Supplementation of CM from donor cells resulted in transfer of miR-424 to the recipient RWPE-1 and LNCaP cells (Fig. 2C). The same was observed when RWPE-1 cells transiently transfected with premiR-424 were used as donor cells. Donor RWPE-1 cells exhibited a high level of mature miR-424 compared to control cells (Fig. 2D) and the CM from these cells (CM-miR-424) increased miR-424 level in recipient cells (Fig. 2E). Notably, transfer of CM containing miR-424 to recipient cells significantly enhanced tumor-sphere formation (Fig. 2F–H), clonogenic capability (Fig. 2G) and cell migration (Fig. 2I) compared to control CM.

**Fig. 3:**
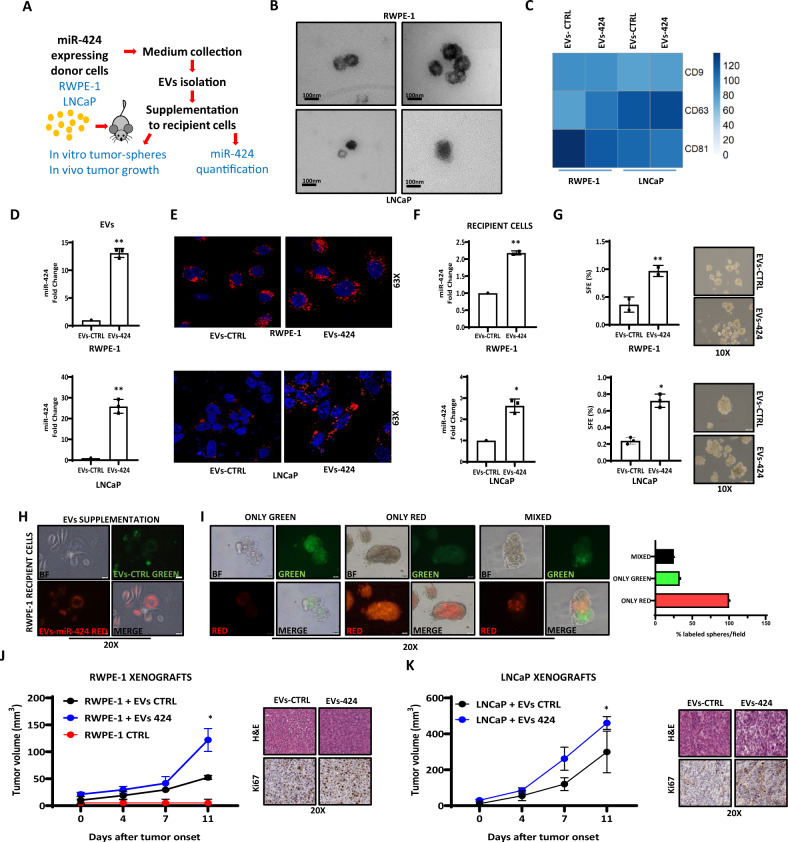
EVs-secreted miR-424 drives the acquisition of stem cell traits and in vivo tumorigenesis. **A** Schematic of EVs isolation and functional characterization. **B** Representative image of transmission electron microscopy (TEM) of cell line-derived EVs. **C** EV-surface markers in cell line-derived EVs by MACSPlex analysis. **D** miR-424 levels in EVs derived from RWPE-1 and LNCaP cells with (EVs-424) and without (EVs-CTRL) stable expression of miR-424. **E** Visualization of recipient cells incubated with fluorescently labeled EVs (PKH26, red) from RWPE-1 and LNCaP donor cells by confocal microscopy. Nuclei were stained with Hoechst (blue). **F** Level of miR-424 in RWPE-1 (upper) and LNCaP (lower) recipient cells incubated with EVs-CTRL or EVs-424. **G** Tumor-sphere formation by RWPE-1 (upper) and LNCaP (lower) cells supplemented with EVs-CTRL or EVs-424. **H** Confocal microscopy images of RWPE-1 recipient cells incubated for 24 h with EVs-CTRL or EVs-424 labeled with PKH67 (green) or PKH26 (red) fluorescent dyes. **I** Confocal microscopy images of tumor-spheres formed by RWPE-1 cells incubated with fluorescently labeled EVs as above. Right: quantification of fluorescently labeled tumor-spheres. **J** Growth of subcutaneous xenografts of RWPE-1 cells supplemented in vitro with EVs-CTRL and EVs-424 derived from stably expressing donor LNCaP cells. *n* = 4/group. **K** Tumor growth of LNCaP cells supplemented in vitro with EVs-CTRL and EVs-424 derived from stably expressing LNCaP donor cells. *n* = 4/group. Right panels, H&E and IHC staining for Ki67 in xenograft explants.*p ≤ 0.05, **p ≤ 0.01 by two-tailed Student’s *t*-test.

Next, we verified whether this involved the release of miR-424-loaded EVs from donor cells and their transfer to recipient cells (Fig. 3A). Small EVs were isolated from CM of both control and miR-424 expressing cells. The isolated EVs exhibited the expected morphology and size (<150 nm) as revealed by TEM and NTA (Fig. 3B and Supplementary Fig. 2A). MACS-PLEX (Fig. 3C) and immunoblotting (Supplementary Figs. 2B and 10 and 11) also showed the presence of canonical EVs markers. Importantly, miR-424-expressing cells secreted EVs with high miR-424 content (Fig. 3D). Following supplementation, fluorescently labeled EVs from both control and miR-424-expressing cells were taken up by recipient cells (Fig. 3E) and the miR-424-loaded EVs transferred efficiently the miRNA into recipient cells (Fig. 3F). Importantly, miR-424 was internalized within EVs, protected by the EVs phospholipid membrane from degradation, as shown by its resistance to RNase treatment (Supplementary Fig. 3). Supplementation of EVs from miR-424 positive (EVs-424) to RWPE-1 and LNCaP cells significantly increased tumor-sphere formation compared to EVs from negative control (EVs-CTRL) cells (Fig. 3G), reproducing the effects of CM.

To confirm that miR-424-loaded EVs promoted tumor-sphere formation, we differentially labeled miR-424 positive (EVs-424) and negative (EVs-CTRL) EVs with either red (PKH26) or green (PKH67) fluorescent dyes. Labeled EVs were mixed in equal amounts and supplemented to recipient cells. Retention of the labeled EVs in the progeny of tumor-sphere cells would indicate the source of the EVs that they had received. Immediately after supplementation, recipient cells showed both red and green fluorescence, indicating equal distribution and uniform intake of the stained EVs (Fig. 3H). At the end of the assay, we found a significant enrichment of red-stained tumor-spheres (Fig. 3I), consistent with the notion that miR-424-loaded EVs had greater ability to promote tumor-sphere formation. To determine whether the acquisition of stem-like traits by miR-424 containing EVs led to increased tumorigenesis, RWPE-1 cells were incubated with EVs-CTRL or EVs-424 in vitro and then were implanted subcutaneously in mice. EVs-424-treated RWPE-1 cells formed larger tumors (≥100 mm^3^) compared to EVs-CTRL treated and untreated RWPE-1 cells (Fig. 3J). Consistently, LNCaP cells supplemented with EVs-424 generated bigger tumors than EVs-CTRL-treated LNCaP cells (Fig. 3K). Thus, supplementation of miR-424-containing EVs promoted tumor-forming capability in immortalized prostate epithelial cells RWPE-1 and enhanced in vivo tumorigenic potential of prostate cancer cells LNCaP.

### Secretion of miR-424 in EVs is enhanced in the transition from indolent to aggressive phenotype in experimental prostate cancer models

**Fig. 4:**
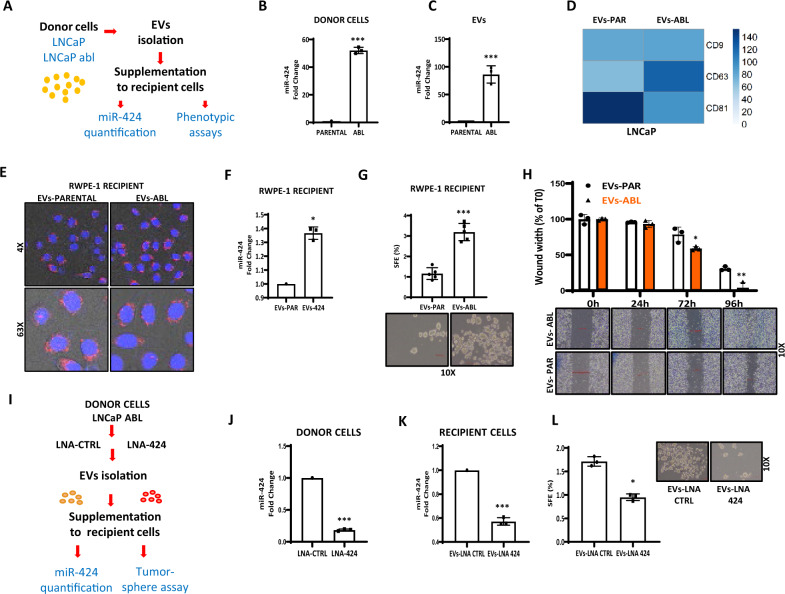
EVs secretion of miR-424 is enhanced during the transition from indolent to aggressive phenotype in experimental models. **A** Isolation and assessment of EVs in LNCaP and LNCaP^abl^ cells. **B** miR-424 expression in LNCaP and LNCaP^abl^ cells. **C** miR-424 levels in EVs derived from parental LNCaP and LNCaP^abl^ cells. **D** Surface markers in EVs from LNCaP (EVs-PAR) and LNCaP^abl^ (EVs-ABL) cells. **E** Confocal images of RWPE-1 recipient cells incubated for 48 h with PKH26-labeled EVs (red) from LNCaP and LNCaP^abl^ cells. Nuclei were stained with Hoechst (blue). **F** Level of miR-424 in recipient RWPE-1 after supplementation of EVs from parental (EVs-PAR) or LNCaP^abl^ (EVs-ABL) cells. **G** Tumor-sphere formation by RWPE-1 cells supplemented with EVs from parental (EVs-PAR) or LNCaP^abl^ (EVs-ABL) cells. **H** Cell migration by wound healing assay in recipient RWPE-1 cells incubated with EVs from parental (EVs-PAR) or LNCaP^abl^ (EVs-ABL) cells. **I** Schematic of the experimental blockade of miR-424 transfer through EVs in LNCaP^abl^ cells. **J** Level of miR-424 in LNCaP^abl^ donor cells transfected with LNA-CTRL or LNA-424. **K** Level of miR-424 in recipient RWPE-1 cells supplemented with EVs from control (EVs-LNA CTRL) or LNA-transfected (EVs-LNA 424) donor cells. **L** Tumor-sphere formation by RWPE-1 recipient cells supplemented with EVs-LNA CTRL or EVs-LNA 424. *p ≤ 0.05, **p ≤ 0.01, ***p ≤ 0.005 by two-tailed Student’s *t*-test.

To support the association between miR-424 upregulation and acquisition of aggressive traits, we examined the expression of miR-424 and release into EVs in LNCaP^abl^ cells, a CRPC cell model derived from LNCaP cells by continuous growth in androgen-depleted medium^16^ (Fig. 4A). First, we found significantly higher expression of miR-424 in LNCaP^abl^ compared to parental cells, in line with a link between miR-424 upregulation and progression to CRPC (Fig. 4B). Furthermore, LNCaP^abl^ cells secrete EVs significantly enriched of miR-424 compared to parental cells (Fig. 4C). Characterization of EVs from LNCaP^abl^ cells by NTA, MACS-PLEX, and immunoblot, indicated that were similar in size to those from parental LNCaP cells and presented similar EVs-specific markers (Fig. 4D and Supplementary Figs. 4A, B and 12). Thus, elevation of miR-424 occurred during the transition to the CRPC phenotype and was associated with a concomitant enrichment of miR-424 in EVs secreted from LNCaP^abl^ cells.

Next, we supplemented RWPE-1 recipient cells with EVs from parental and LNCaP^abl^ cells. We found similar uptake of EVs in recipient cells using parental and LNCaP^abl^-derived EVs (Fig. 4E). However, LNCaP^abl^-derived EVs increased miR-424 level (Fig. 4F) and concomitantly enhanced tumor-sphere formation (Fig. 4G), and cell migration (Fig. 4H) in recipient cells compared to EVs from parental LNCaP cells. These data further supported the potential impact of miR-424 in the prostate cancer evolution. Interestingly, pretreatment of recipient cells with Bafilomycin A1, which blocks the intracellular release of the EVs cargo, prevented the increase in tumor-sphere formation following supplementation with exosome from LNCaP^abl^ cells, indicating that the EVs cargo delivery occurred through the endosomal pathway (Supplementary Fig. 5). To better understand the role of miR-424 as a key mediator of the phenotypes induced by EVs secreted from LNCaP^abl^ cells, we ablated miR-424 in LNCaP^abl^ cells, isolated EVs and supplemented them to recipient cells to evaluate tumor sphere formation (Fig. 4I). Pre-treatment with locked nucleic acid (LNA)-424, a specific miR-424 antagonist^14^, significantly reduced miR-424 level in LNCaP^abl^ cells (Fig. 4J). Consequently, EVs supplementation to recipient cells resulted in lower level of miR-424 intake (Fig. 4K), and reduced tumor-sphere formation compared to EVs from LNA-control transfected cells (Fig. 4L). These data demonstrated that EVs from LNCaP^abl^ cells promoted stem-like features in recipient cells by releasing miR-424 and support a role of miR-424 in disease progression.

### Secretion of miR-424 in EVs from aggressive mouse models and human prostate tumors

**Fig. 5:**
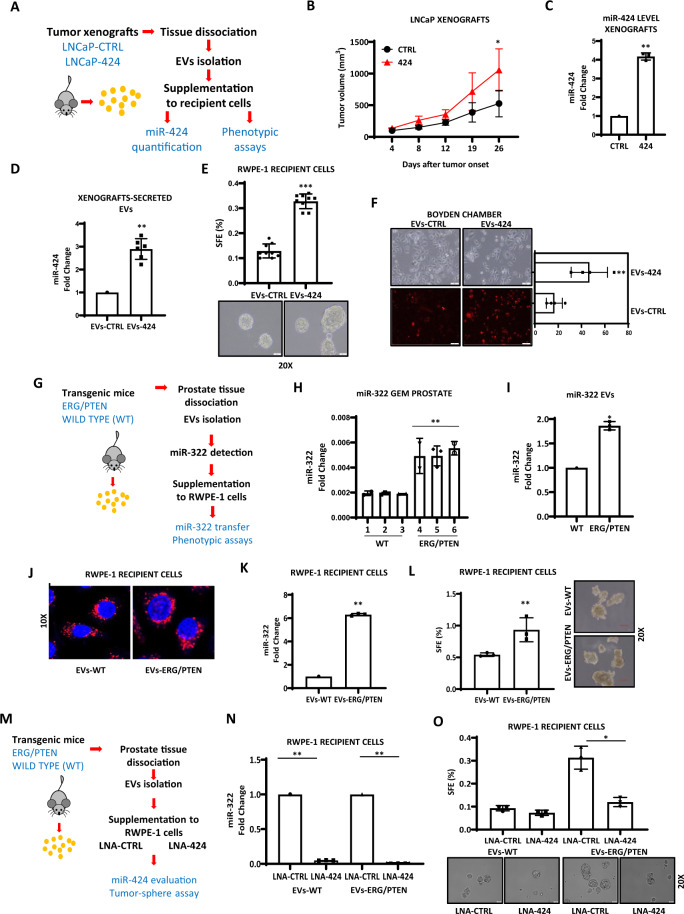
Release of miR-424 containing EVs from human tumor xenografts and genetic mouse models. **A** Assessment of EVs release from human tumor xenografts. **B** Growth of subcutaneous tumor xenografts of parental (CTRL) and miR-424-expressing LNCaP (424) cells in NSG mice. *n* = 4/group. **C** miR-424 level in explants of CTRL and 424 tumor xenografts. **D** miR-424 level in EVs isolated from xenografts (*n* = 2/group) of parental (EVs-CTRL) and miR-424-expressing LNCaP (EVs-424) cells. **E** Tumor-sphere forming assay with RWPE-1-recipient cells incubated with EVs-CTRL and EVs-424 xenograft-derived EVs. **F** Cell migration by Boyden chamber assay with RWPE-1 cells incubated with PKH26-labeled EVs-CTRL and EVs-424 from tumor xenografts. Left, representative phase-contrast and fluorescence microscopy images of RWPE-1 cells. **G** Schematic plan to evaluate EVs release from transgenic mouse models and functional characterization. **H** miR-322 levels in prostatic tissue from wild type (WT) and ERG/PTEN transgenic mice. *n* = 3/group. **I** miR-322 content in EVs isolated from prostatic tissue from WT and ERG/PTEN mice. *n* = 3/group. **J** Confocal images of RWPE-1 cells incubated for 48 h with EVs derived from WT (EVs-WT) and ERG/PTEN (EVs-ERG/PTEN) murine prostates. EVs were labeled with PKH26 (red) and supplemented to recipient cells. EVs (red). Nuclei are stained with Hoechst (blue). **K** miR-322 level in recipient RWPE-1 cells incubated with EVs-WT or EVs-ERG/PTEN. **L** Tumor-sphere assay with RWPE-1 cells supplemented with EVs-WT or EVs-ERG/PTEN. **M** Schematic of the experimental plan to block miR-424 in recipient cells. **N** Level of miR-322 evaluated in recipient cells supplemented with EVs from the indicated GEM source, following blockade of miR-424/322 using LNA-CTRL and LNA-424. **O** SFE in RWPE-1 recipient cells transfected with LNA-CTRL and LNA-424 and subsequent supplementation with EVs from the indicated GEM sources. *p ≤ 0.05, **p ≤ 0.01, ***p ≤ 0.005 by two-tailed Student’s *t*-test.

To examine the release of miR-424 into small EVs from prostate tumors, we established subcutaneous xenografts of control and miR-424-positive LNCaP cells in mice (Fig. 5A). As expected, tumors of miR-424 expressing LNCaP cells grew larger than control tumors (Fig. 5B) and retained high expression of miR-424 (Fig. 5C). Next, EVs were isolated from tumor explants according to a published protocol^17^. Small EVs isolated from miR-424-positive tumors contained high amounts of miR-424 compared to control xenografts (Fig. 5D). Furthermore, tumor-derived EVs were fully functional and promoted tumor-sphere formation (Fig. 5E) and cell migration (Fig. 5F), when supplemented to miR-424-negative recipient cells. Thus, tumor xenografts in mice secrete functional EVs that transfer miR-424 and induce malignant phenotypes in recipient cells.

To gain further evidence of tumor release of miR-424-loaded EVs and the association with aggressive phenotype, we used a genetically engineered mouse model (GEMM) of aggressive prostate cancer^18^ (Fig. 5G). Pb-Cre4;Pten^flox/flox^;Rosa26^ERG/ERG^ (ERG/PTEN) mice combine conditional prostate-specific PTEN deletion and ERG overexpression and develop invasive prostate adenocarcinomas^18^. Notably, we found that prostate tumors of ERG/PTEN mice express higher level of miR-322, the murine orthologous of miR-424, than prostatic tissue from wild type (WT) mice (Fig. 5H). Consistently, small EVs from murine ERG/PTEN tumors had higher content of miR-322 (Fig. 5I). EVs isolated from ERG/PTEN and WT mice were equally taken up by RWPE-1 cells (Fig. 5J). Notably, RWPE-1 cells receiving ERG/PTEN-derived EVs had significantly higher level of miR-322 (Fig. 5K) and enhanced ability to form tumor-sphere (Fig. 5L) compared to cells treated with WT prostate-derived EVs. To support the role of miR-424 in this process, we transfected the LNA antagonist of miR-424/miR-322 in RWPE-1 cells receiving EVs from ERG/PTEN (EVs-ERG/PTEN) and WT (EVs-WT) mice (Fig. 5M). The LNA anti-miRNA significantly reduced the level of miR-322 in recipient cells (Fig. 5N) and reversed the effect of EVs-ERG/PTEN on tumor-sphere formation (Fig. 5O).

**Fig. 6:**
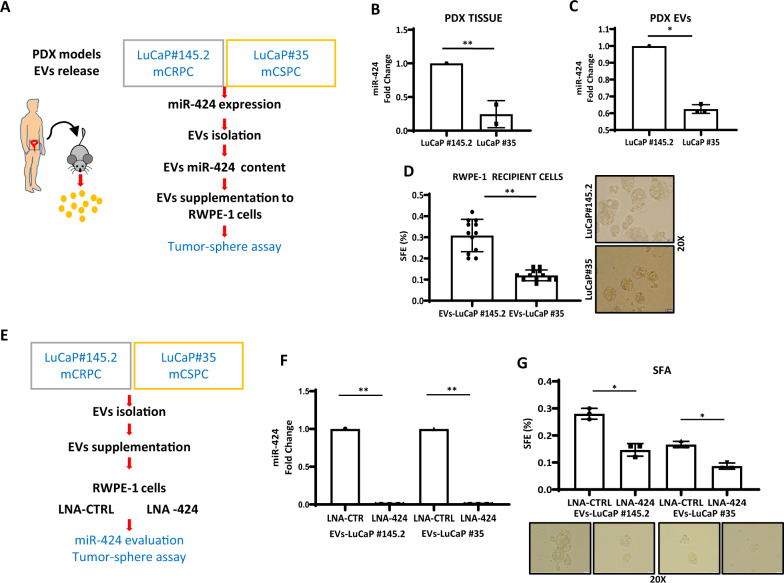
EVs secretion of miR-424 is enhanced in aggressive prostate PDX and promote stemness in prostate epithelial cells. **A** Schematic plan to evaluate EVs release from LuCaP/PDX models and functional characterization. **B** miR-424 level in explants of LuCaP #145.2 (mCRPC) and LuCaP#35 (mCSPC) tumor xenografts (*n* = 2/group). **C** Level of miR-424 evaluated in EVs derived from indicated PDX. **D** Tumor-sphere forming assay with RWPE-1 recipient cells incubated with mCRPC and mCSPC xenografts-derived EVs. **E** Schematic of the experimental plan to block miR-424 in recipient cells. **F** Level of miR-424 evaluated in recipient cells supplemented with EVs from the indicated PDX sources, following blockade of miR-424 using LNA-CTRL and LNA-424. **G** SFE in RWPE-1 recipient cells transfected with LNA-CTRL and LNA-424 and subsequent supplementation with EVs from the indicated PDX sources.*p ≤ 0.05, **p ≤ 0.01.

To support the association between aggressive phenotypes and miR-424 secretion in prostate cancer, we examined two PDX models (Fig. 6A). Both PDXs have been extensively characterized^19^ and represent valid models of mCSPC (LuCaP #35) and mCRPC with neuroendocrine features (LuCaP #145.2), respectively (Supplementary Fig. 6A, B). We found that LuCaP #145.2 xenografts had higher level of miR-424 compared to LuCaP #35 tumors (Fig. 6B). Consistently, EVs released by LuCaP #145.2 had higher content of miR-424 (Fig. 6C). Moreover, when LuCaP #145.2-derived EVs were supplemented to recipient RWPE-1 cells, we observed enhanced tumor-sphere formation compared to LuCaP #35-derived EVs (Fig. 6D).

To demonstrate the contribution of miR-424, we ablated miR-424 in EVs receiving RWPE-1 cells using anti-miR-424 LNA (Fig. 6E). Relevantly, the anti-miRNA LNA reduced the level of miR-424 in recipient cells (Fig. 6F) and accordingly prevented any increase in tumor-sphere formation promoted by the PDX-derived EVs (Fig. 6G). Collectively, these findings supported the oncogenic role of miR-424 in prostate cancer. Furthermore, our data linked secretion and transfer of miR-424 through EVs to aggressive tumor phenotypes, suggesting a role of this process in disease progression to metastatic and hormone-refractory prostate cancer. In keeping with this hypothesis, we found that human CRPCs exhibited enhanced transcriptional activation or repression of genes known to be associated with miR-424 upregulation in primary prostate tumors^14^ (Supplementary Fig. 7A, B). Furthermore, these miR-424-associated gene signatures distinguished clearly CRPCs from primary tumors in unsupervised clustering (Supplementary Fig. 7A, B) and principal component analysis (Supplementary Fig. 7C, D).

### miR-424-loaded circulating EVs are oncogenically active in mouse models and human patients

**Fig. 7:**
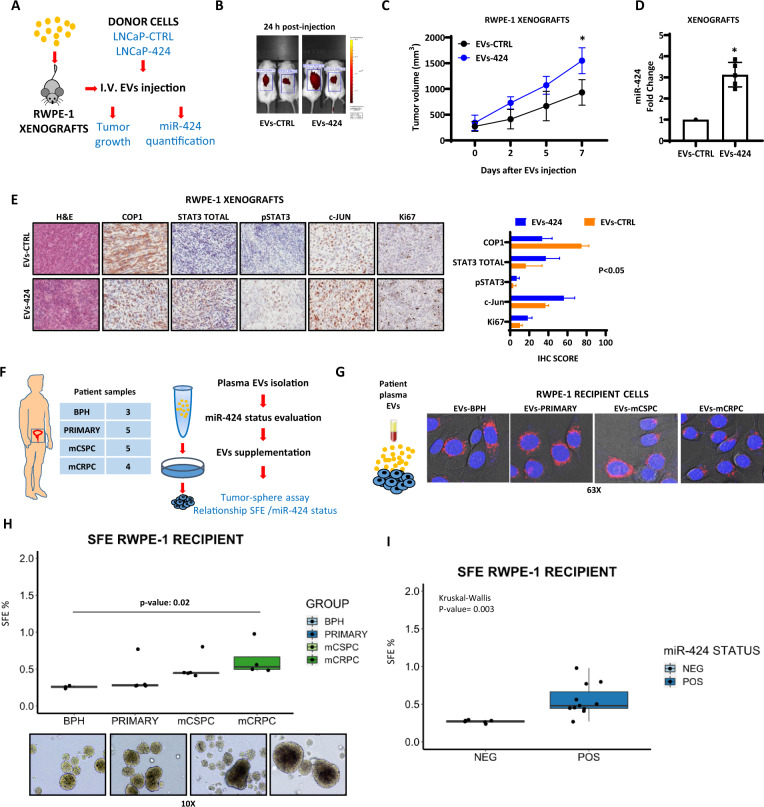
Circulating miR-424-loaded EVs are oncogenically active in mouse models and human patients. **A** Schematic plan for in vivo assessment of EVs functionality. **B** In vivo imaging of distribution of DiD-labeled EVs from control (EVs-CTRL) and miR-424 expressing (EVs-424) donor cells 24 h after intravenous injection to NSG mice with RWPE-1 xenografts. **C** Growth of RWPE-1 xenografts following single tail vein injection of EVs-CTRL and EVs-424 (*n* = 4/group). **D** miR-424 level in RWPE-1 xenografts in mice injected with EVs-CTRL and EVs-424. *E* Assessment of protein markers by IHC in RWPE-1 xenografts from mice injected with EVs-CTRL and EVs-424. Right, IHC scores for each protein markers. **F** Functional assessment of patient-derived EVs in recipient RWPE-1 cells. **G** Confocal microscopy images of RWPE-1 cells supplemented with patient-derived EVs stained with PKH26 (red). **H** Tumor-sphere forming assay of RWPE-1 cells supplemented with EVs derived from the indicated patient groups. Bottom, representative images of tumor-spheres. **I** Tumor-sphere forming efficiency of recipient cells as in **H** shown in relation to miR-424 status after supplementation of patient-derived EVs. *p ≤ 0.05, **p ≤ 0.01 by two-tailed Student’s *t*-test. Following on reports that miR-424 expression promotes oncogenesis, Domenico Albino et al. find that extracellular vesicles (EVs) in the plasma of prostate cancer patients secrete miR-424. Using cell-based and animal models, they demonstrate that EV-mediated release of miR-424 can transfer oncogenic signals across cells to promote recurrence and metastatic progression.

Our data suggested that small EVs or exosomes released by tumors could educate low tumorigenic cells to acquire stem-like and tumorigenic traits both locally and at distal metastatic sites. To test the latter point, we evaluated whether miR-424-loaded EVs given systemically by tail vein injection could circulate in blood and reach the tumor sites to release their cargo (Fig. 7A). Mice with subcutaneous implants of RWPE-1 cells (≥100 mm^3^) received injections of control (EVs-CTRL) and miR-424 loaded (EVs-424) EVs derived from LNCaP cells. Using DiD-labeling and in vivo imaging, we detected fluorescently labeled EVs in the tumor area at 24 h post-injection (Fig. 7B), indicating that both EVs preparations reached the tumors. Importantly, supplementation of EVs-424 enhanced tumor growth compared to EVs-CTRL (Fig. 7C) and reproduced the phenotypic changes previously associated with miR-424 upregulation^14^. Consistently, the level of miR-424 was significantly higher in mice receiving EVs-424 compared to EVs-CTRL (Fig. 7D). The level of COP1, a miR-424 target^14^, was reduced whereas total and phosphorylated STAT3 and c-JUN were increased in EVs-424-treated mice (Fig. 7E). We further confirmed efficient uptake of miR-424 in implanted RWPE-1 xenografts in an independent experiment, in which mice were sacrificed 5 days after receiving injections of EVs-CTRL and EVs-424 and miR-424 quantified in RNA from xenografts by RT-qPCR (Supplementary Fig. 8A, B). These results demonstrated that miR-424-containing EVs could travel through the blood circulation and activated the oncogenic cascade associated with miR-424 in recipient cells at distal sites.

Next, to verify the oncogenic impact of human circulating EVs and their ability to educate low tumorigenic cells to acquire stem-like features, we supplemented RWPE-1 cells with EVs isolated from plasma (*n* = 17) of patients with BPH, primary and metastatic prostate tumors and performed in vitro tumor-sphere assays (Fig. 7F). Using confocal microscopy, we detected similar intake of fluorescently labeled EVs independently of their sources (Fig. 7G). Notably, EVs from mCSPC and mCRPC patients enhanced tumor-sphere formation significantly more than those from patients with BPH and primary tumors (Fig. 7H). Furthermore, high miR-424 content in EVs was significantly associated with increased induction of tumor-sphere formation across all samples (Fig. 7I). These data, therefore, were consistent with the findings in transgenic mice and PDXs described above. Thus, circulating plasma EVs with high miR-424 content could reprogram low tumorigenic cells to acquire stem-like potential and tumorigenic capacity promoting prostate cancer progression.

## Discussion

Given their prevalence and stability in biological fluids, miRNAs can function as efficient inter-cellular signaling molecules acting in autocrine, paracrine, or endocrine manner^6,8,20,14^. In this study, we show for the first time that patients with advanced prostate cancer release fully functional circulating EVs containing miR-424, which facilitate the acquisition of stem-like traits by low tumorigenic cells and thus contribute to disease propagation and progression. We provide several lines of evidence for the release of miR-424 into small EVs and its efficient intake by recipient cells. Using various experimental models, from cell cultures to PDXs and genetic mouse models, we show that EVs-mediated transfer of miR-424 propagates the oncogenic signal promoting stem-like features like in vitro tumor-sphere capability and in vivo tumor growth. Furthermore, mimicking the action of circulating EVs in cancer patients, we demonstrate that miR-424-loaded EVs injected intravenously transfer efficiently their cargo to subcutaneously implanted recipient cells and promote growth of the xenografts in mice. Interestingly, in line with a relation between miR-424 and tumor aggressiveness, we found increased expression and EVs-mediated release of miR-424 in LNCaP^abl^ cells, ERG/PTEN mice and PDX LuCaP #145.2, models of hormone-refractory or invasive adenocarcinomas. Consistently, miR-424-loaded EVs were preferentially detected in plasma of patients with metastatic tumors with low or undetectable levels in primary tumors and BPH. Moreover, patient-derived miR-424-positive EVs were fully functional and their ability to promote stemness, a feature associated with increased in vivo tumorigenic and metastatic capability, was directly related with the clinically advanced stage and content of miR-424. Future studies in larger cohorts of patients at various stages of the disease will determine the validity of miR-424-loaded EVs as biomarker for the management of prostate cancer and investigate their relation with other molecular, biological, and clinical features in prostate tumors. Taken together, our findings indicate that release of miR-424 in EVs is a property associated with advanced and aggressive prostate tumors and can facilitate disease recurrence and progression by allowing rapid transfer of oncogenic signals among phenotypically heterogeneous tumor cell subpopulations. As miR-424-loaded EVs can be easily detected in the patient plasma, their measurement could be used as a minimally invasive test to identify patients at risk of progressive and metastatic disease. Furthermore, we show that a miR-424 antagonist is effective in limiting the release of miR-424 in EVs and the functional consequences in recipient cells, suggesting that this horizontal pathway of inter-cellular transfer of oncogenic signals may offer novel targets for therapeutic intervention and drug discovery.

## Methods

### Human samples

Blood (3–6 mL) was collected from patients with BPH (*n* = 6), primary (*n* = 25), metastatic castration sensitive (mCSPC) (*n* = 16), and metastatic castration-resistant prostate cancer (mCRPC) (*n* = 17), between 2014 and 2019 at the Portuguese Oncology Institute of Porto (Porto, Portugal), Groote Schuur, Eerste Rivier and New Somerset Hospitals (Cape Town, South Africa) and Oncology Institute of Southern Switzerland (Bellinzona, Switzerland). Samples were collected prior to any treatment at the time of diagnosis or during routine follow-up for metastatic patients. Metastatic disease was defined by radiologic progression, whereas CRPC was defined as previously described^21^.

### Plasma collection and processing storage

Plasma was separated by centrifugation (2880 × *g*, 10 min, 4 °C) from peripheral blood collected in EDTA tubes. Samples were stored at −80 °C at the Institutions biobanks. Ethical approval for the study was obtained by the ethical committees of the individual collaborating Institutions: Comissão de Ética para a Saúde (CES-IPOFG-EPE 205/2013, IPO Porto), Human Research Ethics Committee (HREC454/2012, University of Cape Town), and Ethical Committee of Canton Ticino (CE TI 3269 Project-ID 2017–01631, IOSI). Clinical and pathological data (Supplementary Data 1) were collected in an anonymized database.

### Isolation of small EVs from human plasma

EVs were isolated from 1.5 to 2 mL of patient-derived plasma samples using the Total Exosome Isolation Kit (Catalog Number: 4484450, Invitrogen) according to the protocol. Plasma was centrifuged (2000 × *g*, 20 min, RT) to remove cells and debris. Supernatant was transferred to clean tube and centrifuged again (10,000 × *g*, 20 min, RT) to remove further debris. Pellets were resuspended with 100 µL of PBS and stored at −80 °C until use. Aliquots of intact EVs were used for protein quantification using bicinchoninic acid assay (BCA). Isolated EVs were stored at −20 °C until use. For miR-424 evaluation isolated EVs were resuspended in 100 µL of PBS and used for Directzol RNA extraction (see “RNA extraction, RT-qPCR, and miRNAs expression analysis”).

### EVs isolation from cell medium

Conditioned medium (CM) (3–5 mL) was collected from cells incubated for 48 h and maintained in their standard medium with EV-depleted FBS. EVs were isolated using miRCURY Exosome isolation kit (Cells, Urine and CSF, EXIQON, cat. #300102). CM was centrifuged (3000 × *g*, 10 min, RT) to remove cells and debris. We standardized the EVs collection and quantification by using the following steps: 3–5 × 10^6^ donor cells were seeded in 3–5 mL in a T25 flask and after 48 h the EVs were isolated using the miRCURY Exosome isolation kit. After resuspending the intact EVs pellets in 100 µL of PBS, an aliquot of 20 µL was used for lysis in RIPA buffer 2× with protease inhibitor cocktail (Roche) and phosphatase inhibitor cocktail (PhosStop; Roche); an aliquot of 20 µL was used for BCA assay for protein quantification. To isolate larger amounts of EVs, we used the differential ultracentrifugation (DUC) approach^22^. Specifically, cells were cultured in EV-depleted medium at the concentration of 24 × 10^6^ cells/flask. After 48 h, 30 mL of conditioned medium was centrifuged (300 × *g*, 10 min, 4 °C). The supernatant was recovered and centrifuged (2000 × *g*, 10 min, 4 °C). The recovered supernatant was centrifuged (10,000 × *g*, 30 min, 4 °C). The supernatant was ultracentrifuged by using Beckman LE-80K (100,000 × *g*, 2 h, 4 °C) using a SW32.1 rotor (swinging bucket). In this study, both size and external features of the isolated EVs were compatible with the isolation of EVs, according to the minimal information for studies of extracellular vesicles (MISEV 2018) of the International Society of EVs^15^.

### RNA extraction, RT-qPCR, and miRNAs expression analysis

Total RNA extraction was performed using Trizol^®^ (Invitrogen, cat. #TR118) and subsequently by Direct-zol RNA Miniprep kit (Zymo Research, cat. #R2052) that allows the recovery of small RNAs. The RNA quality was verified by the spectrophotometric analysis through the Nanodrop instrument (Thermo Fisher Scientific, cat. #ND-2000). For miRNA expression analysis, 50 ng of purified total RNA was retro-transcribed using TaqMan^®^ MicroRNA Reverse Transcription Kit (Applied Biosystem) with specific primers for the miR-424 (TaqMan^®^ MicroRNA Assays ID: 4427975-000604, Applied Biosystem) and the cDNA was subjected to TaqMan Probe-based real-time PCR (TaqMan^®^ Universal PCR Master Mix, Applied Biosystem). Relative expression of miR-424-5p was calculated using the 2^−ΔΔCT^ method. The miR-424 expression was normalized to RNU6B or miR-21 used as endogenous controls (Control miRNA assay, RNU6B ID: 4427975-001093, miR-21 ID: 4427975-000397, Applied Biosystem). In all the conditions used in our experiments we observed constantly similar Ct values for both small-nuclear ribonucleoprotein (RNU6B) and miR-21. Thus, we used (RNU6B) and miR-21 as endogenous controls for RT-qPCR.

### NTA analysis

The size and concentration of EVs were determined by NTA using a Nanosight NS300 device (Malvern Instruments). Samples were diluted 1:500 in PBS, whereby particle concentration was within the optimal range of detection (5 × 10^7^–1 × 10^9^ particles/mL). Settings were kept fixed during all of the acquisitions in each experiment. For each sample, at least three measurements were taken and analyzed using the NTA software 3.0 with default settings.

### Transmission electron microscopy

For morphological analysis, EVs suspensions (10 μL) were fixed in 50 μL of 2.5% glutaraldehyde and 2% paraformaldehyde in 0.1 M sodium cacodylate, pH 7.6 for 1 h. Five microliters of the mix were transferred onto copper 300 Mesh Formvar-carbon-coated electron microscopy grids (Pacific Grid Tech, www.grid-tech.com). After 20 min, grids were transferred on drops of 50 μL of 0.1 M sodium cacodylate pH 7.6 with the sample membrane side facing down. After three washing in 0.1 M sodium cacodylate, grids were negatively stained with 2% uracyl acetate in water for 10 min and air dried for 5 min. All samples were observed with a Jeol 1010 EX electron microscope (Jeol, Tokyo). Data were recorded with a MORADA digital camera system (Olympus, Tokyo).

### EVs immunophenotype analysis (MACSPlex)

An aliquot of 60 µL of sample underwent bead-based multiplex EVs capture and analysis by flow cytometry (FC), using MACSPlex human Exosome Kit (Miltenyi Biotec; Bergisch Gladbach, Germany), according to manufacturer’s instructions. Briefly, samples were diluted to a final volume of 120 µL with MACSPlex buffer (MPB) and incubated overnight (14–16 h) on an orbital shaker (800 rpm, 10 °C, protected from light) with MACSPlex Exosome Capture Beads, containing 37 antibody-coated bead subsets. MPB was used as blank control. After incubation, 1 mL of MPB was added to each tube and then centrifuged (3000 × *g*, 10 min, 10 °C) to wash beads. After careful aspiration of 1 mL supernatant, 15 µL of MACSPlex Exosome Detection Reagent (5 µL for each allophycocyanin [APC]-conjugated anti-CD9, anti-CD63, and anti-CD81 detection antibody) were added and incubated for 15 min on an orbital shaker (450 rpm, 10 °C), protected from light. After additional washing step, samples were manually mixed and immediately loaded and acquired by MACSQuant Analyzer 10 flow cytometer (Miltenyi Biotec; Bergisch Gladbach, Germany). Median fluorescence intensity (MFI) was evaluated for each capture beads subsets and corrected by subtracting the respective MFI of blank control and normalized by the mean MFI of CD9, CD63, and CD81. Multiplex platform analysis and gating strategy were previously described^23,24^.

### Cell cultures

LNCaP cells were obtained from ATCC and maintained in RPMI-1640 (Gibco) supplemented with 10% fetal bovine serum and 1% of penicillin. Immortalized human prostate epithelial cells RWPE-1 were maintained in keratinocyte serum-free growth medium (KSF; Gibco) with specific supplements^14^. UGSM cells were obtained from ATCC and maintained as previously described^14^. LNCaP/RWPE-1 stably expressing miR-424 (LNCaP/RWPE-1-424) and empty vector (EV) as control (LNCaP/RWPE-1-EV) were established as previously described^14^. LNCAP^abl^ were grown in charcoal-stripped serum (CSS) as previously described^25^.

For miRNA inhibition, cells were transiently transfected for 48 h with 40 nM of a specific LNA antagomiR (Mercury LNA Power Inhibitor; Exiqon) or a scrambled control (Negative Control A; Exiqon). Transfections were performed using Lipofectamine 2000 reagent (Invitrogen) as previously described^14^ and miRNA evaluated as described above. For EVs isolation from transfected cells, 48 h post-transfection CM was collected for EVs isolation as described above. For transient miR-424 overexpression with the miRNA precursor, RWPE-1 cells were transfected for 72 h with 30 nM of miR-424 precursor (miR-424; AM17100-PM10306, Ambion) or negative control #1 (Ctr; AM17110, Ambion). After 72 h, miR-424 was measured by RT-qPCR using TaqMan^®^ MicroRNA Assays as previously described.

### Patient-derived xenografts

LuCaP PDX lines #145.2 and #35 were provided by the laboratory of Prof. Theurillant and were established from specimens acquired at either radical prostatectomy or at autopsy, implanted, and maintained by serial passage in immune-compromised male (NOD.Cg-PrkdcSCID Il2rgtm1Wjl/SzJ (NSG)) mice^19^. All animal experiments were carried out according to the protocol approved by the Swiss Veterinary Authority (TI-42-2018).

### EVs labeling, supplementation in vitro, and confocal analysis

For uptake experiments, EVs were fluorescently labeled by using PKH26 or PKH76 kit from Sigma (MINI26-MINI76). In brief, 5 µg (protein content based) of intact EVs were incubated with 0.4 µL of red dye diluted in 200 µL of diluent C for 5 min. Then, the staining was blocked using 10% EXO-depleted FBS, followed by two centrifugation steps with RPMI-1640. Labeled EVs were used in uptake experiments. RWPE-1 and LNCaP recipient cells (3000 cells/well/200 µL) were seeded in IbiTreat µslide-8well, (IBIDI GmbH, Germany, cat. #80826). After 24 h, labeled EVs were added to recipient cells and incubated at 37 °C for additional 24 h. Before image analysis, Hoechst was added and confocal images were taken. For SFA, after EVs labeling, the equivalent of 8 µg of total EVs-derived proteins, were used for in vitro supplementation to recipient cells. After 48 h, recipient cells were trypsinized and counted by trypan blue and used for in vitro and in vivo experiments. For the tracing of labeled EVs in tumor-spheres forming assay, 1 µg of PKH76 (green) or 1 µg of PKH26 (red)-labeled EVs derived from LNCaP-CTRL and LNCaP miR-424 cells were supplemented to RWPE-1-recipient cells for 48 h. Then, recipient cells were visualized by optical microscope, imaged and tumor-sphere forming assay was performed. After 11 days tumor-spheres were scored by counting red, green, and mixed colored spheres. Images were taken by applying red and/or green filters in phase contrast microscopy.

### Localization of miR-424 in EVs (miRNA protection assay)

EVs containing miR-424 were isolated from RWPE-1 miR424 stable cells and incubated with RNase If (1 µL, 50,000 units/mL, NEB, Ipswich, MA) for 20 min at 30 °C, or with the addition of 1% Triton. Following incubation, RNA was extracted using Direct-zol Miniprep kit as described above.

### Inhibition of EVs cargo release

Recipient RWPE-1 cells were seeded at 1 × 10^6^ cells/well in 2 mL of standard medium. After 24 h, cells were treated with Bafilomycin A1 (100 nM, B1793, Sigma) or DMSO for 30 min^26^. Then, EVs derived from LNCaP^abl^ (5 µg protein content) were supplemented for 48 h and then tumor-sphere forming assay was performed as described above.

### EVs isolation from tissues in tumor xenografts and genetic mouse models

EVs from xenograft tumor tissues were isolated as previously described^17^. Tissue samples were cut into small pieces and incubated in RPMI medium with EXO-depleted FBS for 12–24 h. The medium collected and centrifuged (800 × *g*, 10 min, RT), followed by an additional centrifugation step (20,000 × *g*, 20 min, RT) to remove any residual cellular debris. Next, the supernatant was filtered using 0.2-µm filters and transferred to a 15-mL conical tube. Exosome were isolated using miRCURY Exosome isolation kit and RNA extracted, as described above. To isolate EVs from prostatic tissue from WT and ERG/PTEN mice^27^, prostates were removed from >25-week-old mice, EVs isolated and RNA extracted as described above. Murine miR-424 (miR-322) was evaluated by Thermo Fisher probe (TaqMan^®^ MicroRNA Assays ID: 322001076, Applied Biosystem).

### Tumor-sphere formation, cell migration, and invasion assays

Sphere forming assay was carried out as previously described^14^. Cell migration ability was assessed using scratch/wound-healing (WH) assay as previously described with some modifications^11^. Conditioned medium (6 mL), obtained from RWPE-1 cells transfected for 72 h with Pre-miR-424 or Pre-NEG CTRL, was added to RWPE-1-recipient cells in T25 flask for additional 72 h. Recipient RWPE-1 cells (300,000/well) were grown to confluence in six-well plates for 24 h and a scratch was introduced in the cell monolayer. Pictures of cells were taken at 0, 24, and 48 h using a Zeiss microscope with a Canon EOS 450D camera (×4 magnification). Wound width was shown as percentage relative to time 0.

### Tumor xenografts establishment and in vivo systemic administration of EVs

NSG mice (4–6 weeks old, Jackson Laboratories) were used for in vivo experiments. RWPE-1 cells (2 × 10^6^) along with UGSM2 cells (2 × 10^5^) in a volume of 200 µL (1:1 volume) of RPMI and Matrigel Matrix (BD Biosciences, 354234, low density) were injected subcutaneously in mice (*n* = 4/group).Tumor growth was monitored every 2 days with a caliper and final tumor weight was measured. For in vivo systemic administration, EVs were prepared from control (EVs-CTRL) and miR-424 expressing (EVs-424) donor cells using DUC. To examine in vivo distribution, EVs were labeled with DiD (Biotium, USA, #60014) for 30 min (DiD final concentration 5 mM). After two rounds of centrifugation at 100,000 × *g* for 3 h, EVs were suspended in PBS. EVs (10 µg protein content/mouse in 100 µL of PBS) were injected intravenously in tumor-bearing mice. Total RNA was isolated from xenograft tissues using Tryzol. miR-424 levels were determined by RT-qPCR as described above. All animal experiments were conducted with approval of the Swiss Cantonal Authority (CCEA) and in accordance with national guidelines.

### Immunoblotting

Cell lysates were prepared as previously described^14^ using the following Ab, CD9 (C-4, sc-13118, Santa Cruz Technology) CD81 (EPR4244, ab109201, Abcam), GRP94 (cat. #2104, Cell signalling technology), β-actin (ab8226, Abcam), Calnexin (AF18, cs-23954, Santa Cruz Technology), GAPDH (cat. #0411, sc-47724, Santa Cruz Biotechnology).

### Immunohistochemistry (IHC)

IHC was performed as previously described^14^ using the following Ab: anti-STAT3 (124H6; Cell Signaling; catalog 9139); anti-p-STAT3 Tyr705 (D3A7; Cell Signaling; catalog 9145); anti-COP1 (ab56400, Abcam); anti-c-Jun (E254, ab32137); anti-EZH2 (D2C9, CST#5246); anti-Ki67 (Lab Vision Corp.; ready-to-use RT-9106-R7). The specificity of all antibodies was previously confirmed by Western blot analysis. Cell nuclei were counterstained with hematoxylin solution. Slides were evaluated by at least two investigators in a blinded manner.

### Statistics and reproducibility

In order to assess the differences in miR-424 positive (Ct level < 40) and negative patients (Ct level undefined) among the four patients groups, the Fisher’s exact test was employed. The differences in Ct for miR424 were assessed using the Kruskal–Wallis rank sum test and subsequently the post-hoc Dunn Test for multiple comparisons. The differences in tumor-sphere forming efficiency according to the EVs source and miR-424 status were assessed with the Kruskal-Wallis rank sum test and the post-hoc Dunn Test. In all the tests, the significant threshold was set to 0.05 and the Benjamini–Hochberg correction method was adopted.

### Bioinformatic analysis

Previously identified miR-424-associated gene signatures (including 1806 upregulated genes and 260 downregulated genes^14^ (Supplementary Data 2)), were investigated in a cohort of primary and castration-resistant prostate cancer samples. RNA-Seq datasets, including 497 primary prostate cancers from The Cancer Genome Atlas (TCGA, http://gdac.broadinstitute.org/) and 75 CRPCs (Fred Hutchinson Cancer Research Center) was used. Sequencing reads were aligned as previously described^28^. Heat map plots were generated in R environment. Principal component analysis was performed using the PCAtools (R package version 2.0.0. https://github.com/kevinblighe/PCAtools).

### Reporting summary

Further information on research design is available in the Nature Research Reporting Summary linked to this article.

## Supplementary information

Supplementary Information

Description of Supplementary Files

Supplementary Data 1

Supplementary Data 3

Supplementary Data 2

Reporting Summary

## Data Availability

All data generated and analyzed during the current study are available in Supplementary Data 3. Any remaining data can be obtained from the corresponding author upon reasonable request.
