# Supplementary material for: Circulating extracellular vesicles release oncogenic miR-424 in experimental models and patients with aggressive prostate cancer
Source: Commun Biol. 2021 Jan 26;4:119. doi: 10.1038/s42003-020-01642-5 (PMC7838273; doi:10.1038/s42003-020-01642-5)
Supplement: Supplementary file 1 — Supplementary Information [file 42003_2020_1642_MOESM1_ESM.pdf]

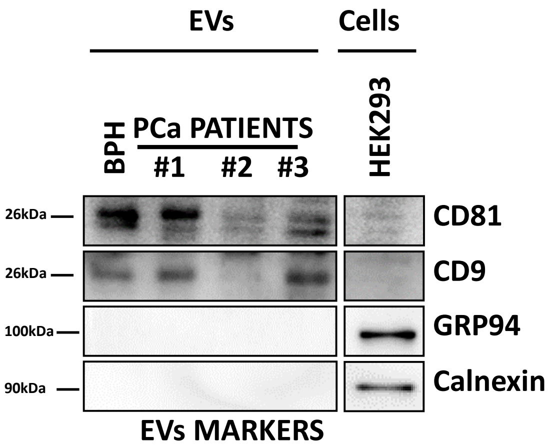

Supplementary Figure 1. Characterization of EVs from plasma of human patients. EVs and cellular protein markers evaluated by immunoblotting in EVs preparations from patient-derived EVs by immunoblotting. HEK293 total cell lysates were used as control.

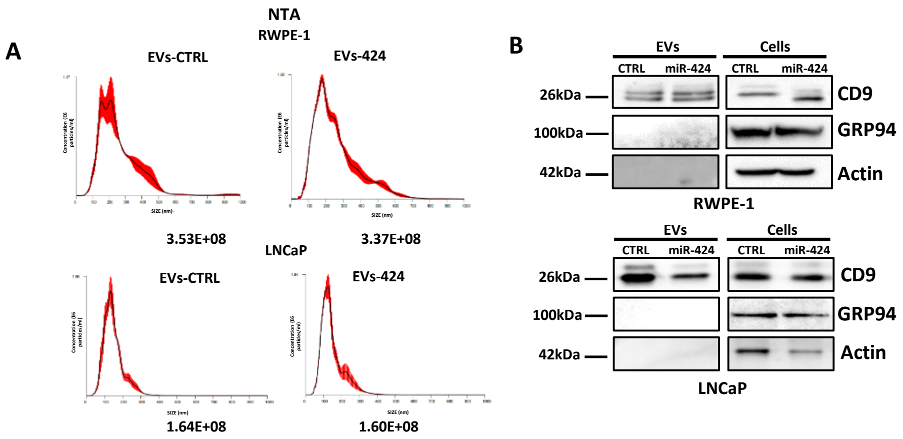

Supplementary Figure 2. A. Size distribution of EVs isolated from RWPE-1 (EVs-CTRL) and (EVs-424) cells (top) and LNCaP (EVs-CTRL) and (EVs-424) cells (bottom) determined by nanoparticle tracking analysis (NTA). B. EVs and cellular protein markers evaluated by immunoblotting in EVs and cell lysate preparations from the indicated cell lines.

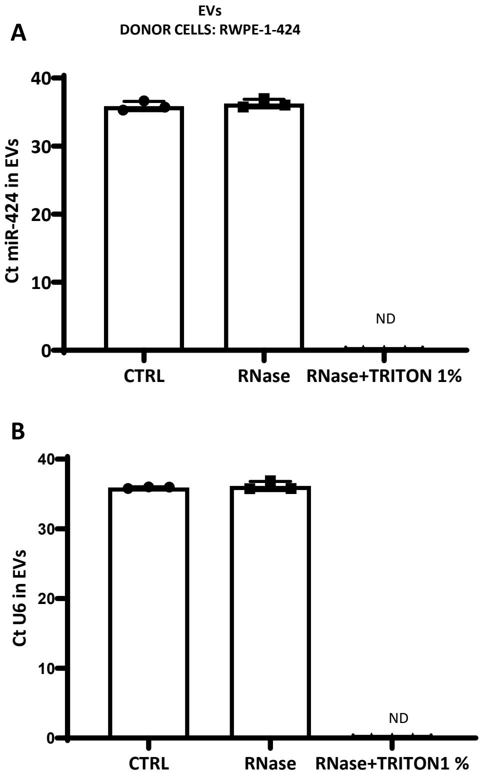

Supplementary Figure 3. miR-424 is located inside the EVs. A-B. EVs were isolated from RWPE-1 (RWPE-1-424) cells and either untreated (CTRL) or treated with RNase or RNase and 1% Triton. RNA was extracted and miRNA-424 (A) and U6 RNA (B) were evaluated by RT-qPCR and Ct values are shown.

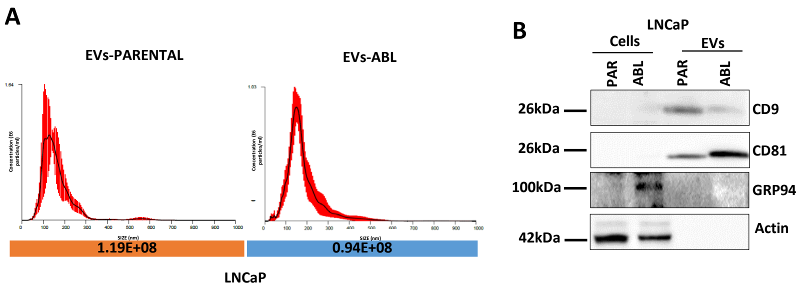

Supplementary Figure 4. **EVs characterization in LNCaP-ABL cells**. A. Size distribution of EVs isolated from LNCaP (EVs-PARENTAL) LNCaP^{abl} (EVs-ABL) cells determined by nanoparticle tracking analysis (NTA). B. EVs and cellular protein markers evaluated by immunoblotting in EVs and total cell lysate preparations from the indicated cell lines.

Supplementary Figure 5

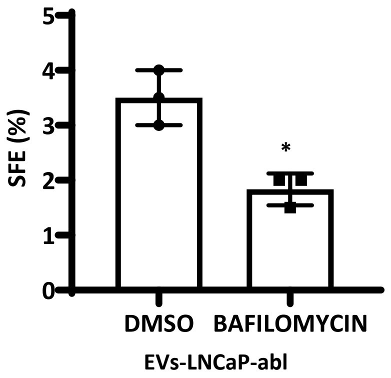

Supplementary Figure 5. Blocking EVs cargo release rescues the impact on recipient cells. Recipient RWPE-1 cells were treated with Bafilomycin A1 (100nM) or DMSO for 30 minutes before supplementation of LNCaP-^{abl} EVs for 48 h. Tumor-sphere formation efficiency (SFE) was assessed.

A

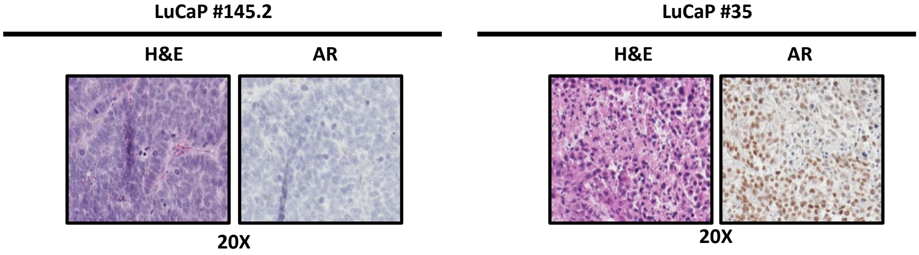

B

	DATE OF TRANSPLANT	TISSUE SOURCE	HISTOLOGY	PSA IN SERUM	AR (IHC)	GROWTH IN BONE	RESPONSE TO CASTRATION
**LuCaP #35**	1993	LN METASTASIS	AC	+	++	YES	+++
**LuCaP #145.2**	2005	LN METASTASIS	NE	-	-	YES	-

Supplementary Figure 6. Histological characterization of LuCaP PDX xenografts. A. IHC representative images of PDX-derived xenografts by H&E and AR. B. Summary of histologic and clinical parameters for PDX-derived xenografts of LuCaP models (described in reference 19).

Supplementary Figure 7

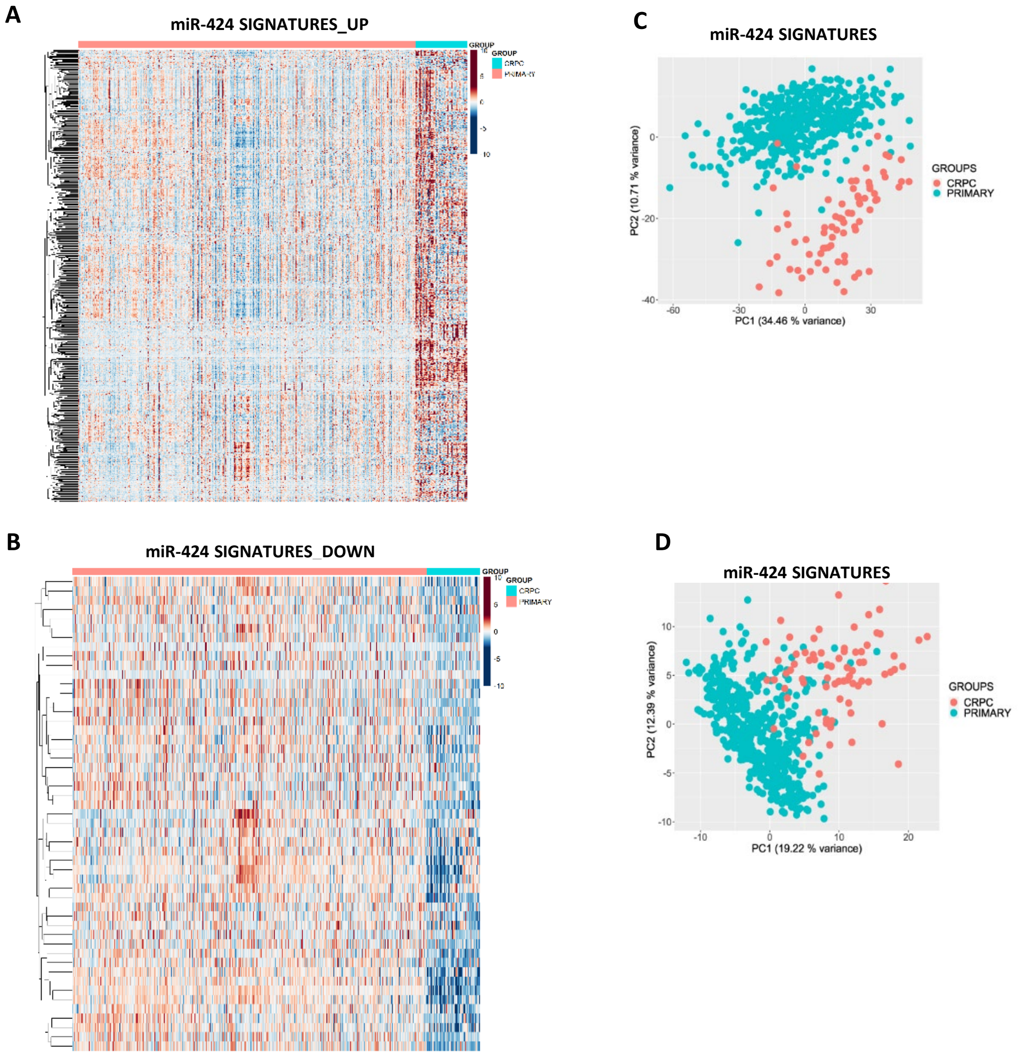

Supplementary Figure 7. miR-424 gene signatures are enriched in CRPC compared to primary prostate tumors. A-B. Heat maps of primary and metastatic prostate tumors using the genes upregulated (A) and downregulated (B) in the miR-424 gene signature (see Material and Methods for details). C-D. Principal component analysis (PCA) of primary and metastatic tumors using the genes upregulated (C) and downregulated (D) from the miR-424 gene signature.

Supplementary Figure 8

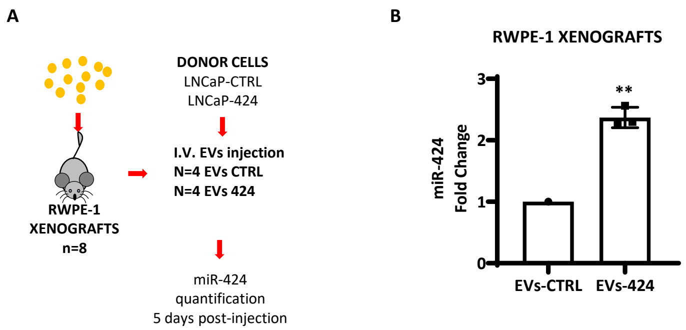

Supplementary Figure 8. Systemic administration of EVs results in efficient delivery of miR-424 in subcutaneous tumors. A. Schematic plan for in vivo assessment of efficient delivery of EVs cargo. RWPE-1 xenografts were established in NSG mice (n=8). Then, mice bearing RWPE-1 xenografts were injected by tail vein with EVs-CRTL and EVs-424. B. Five days post-injection, mice were sacrificed and miR-424 level evaluated in RWPE-1 xenografts (n=4 xenografts/group).

Supplementary Figure 9

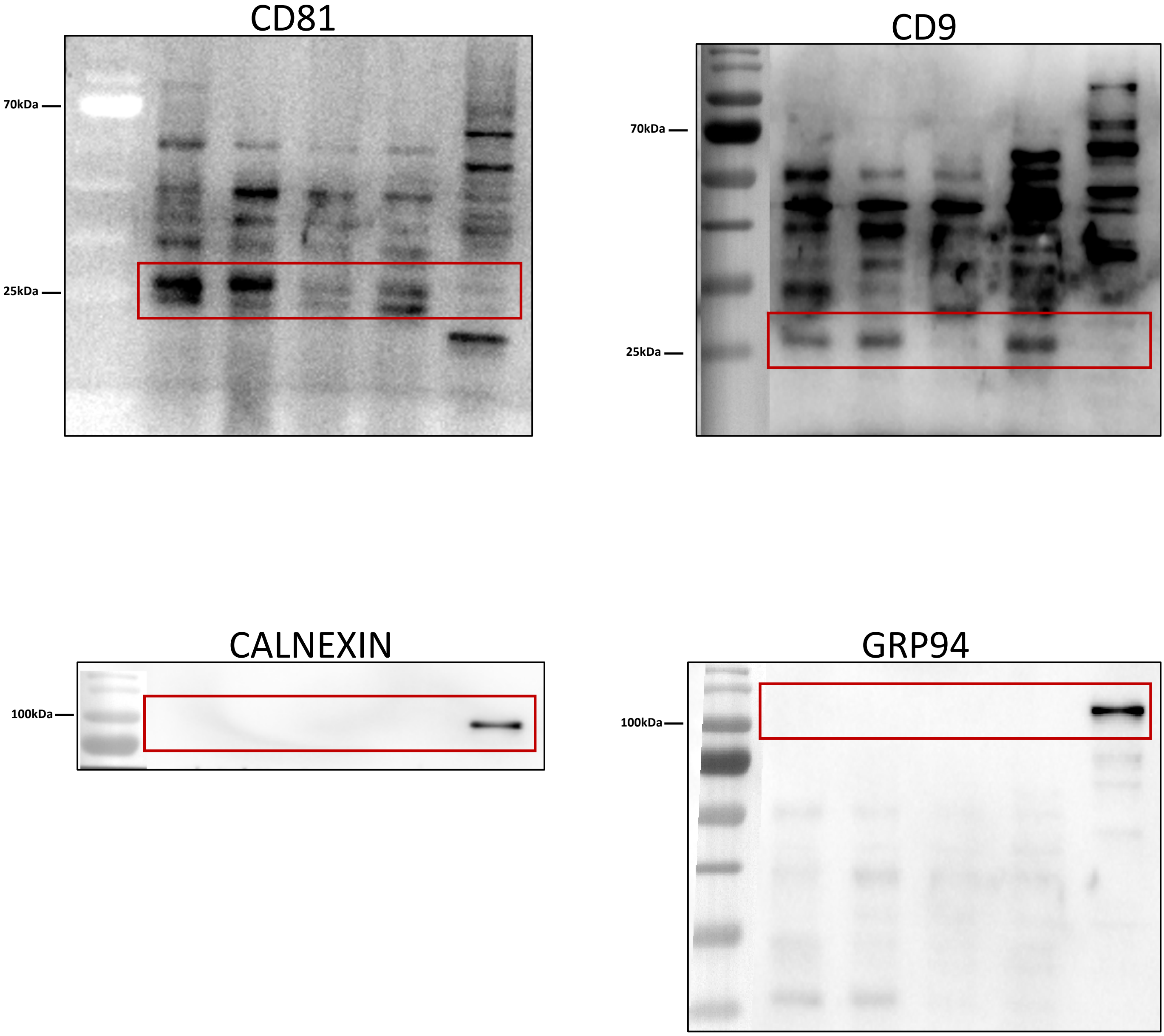

Supplementary Figure 9 (Related to Supplementary Figure 1). Characterization of EVs and cells. EVs and cellular protein markers evaluated by immunoblotting in EVs preparations from patient-derived EVs by immunoblotting. HEK293 total cell lysates were used as control.

CD9

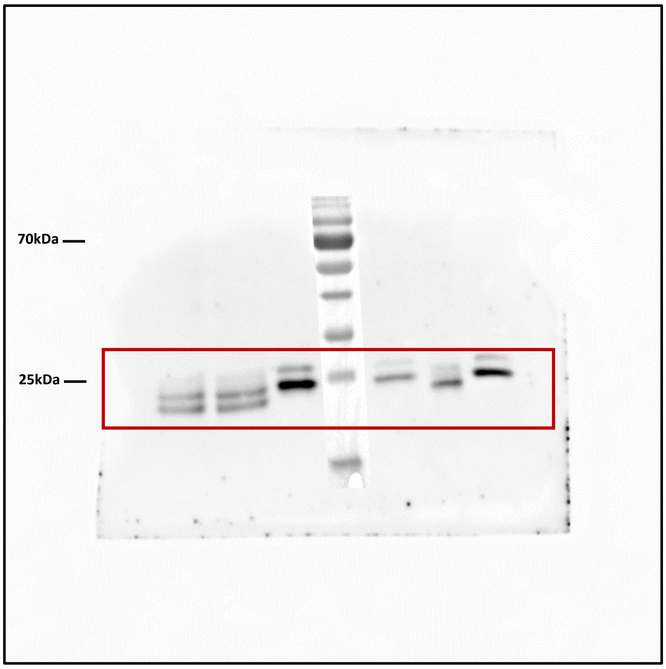

ACTIN

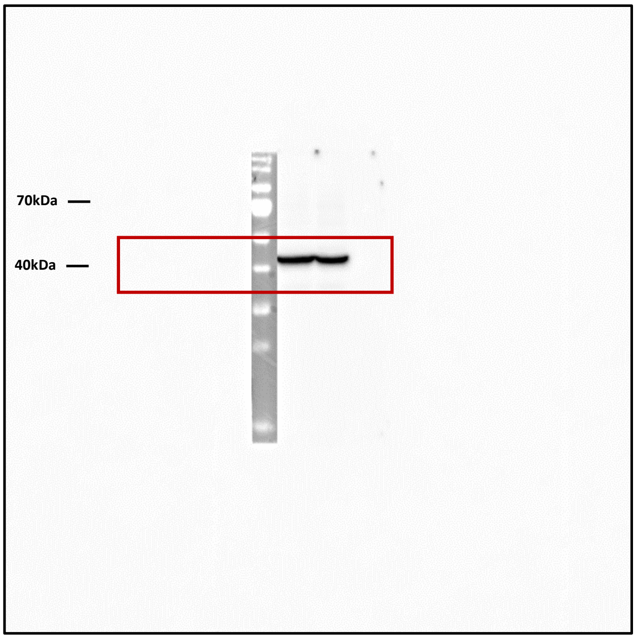

GRP94

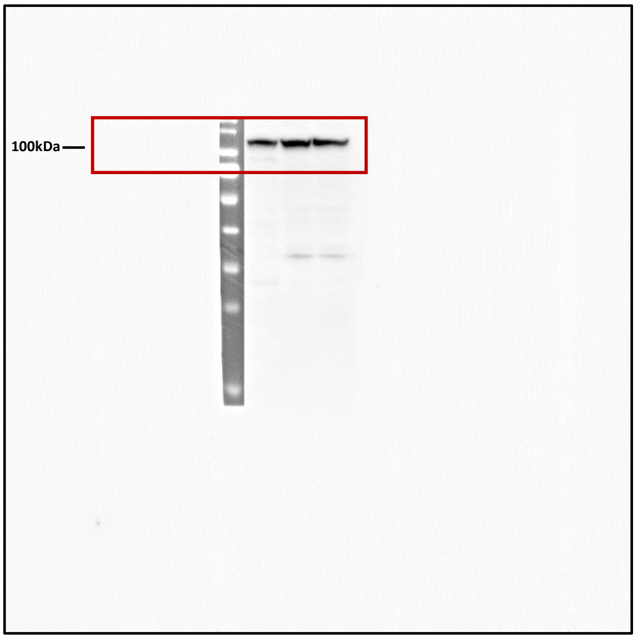

Supplementary Figure 10 (Related to Supplementary Figure 2). Characterization of EVs and cells. EVs and cellular protein markers evaluated by immunoblotting in EVs and cell lysate preparations from RWPE-1 cell lines.

CD9

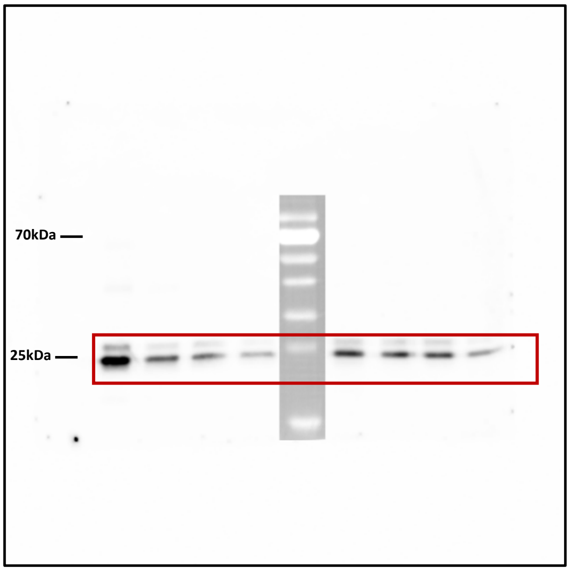

ACTIN

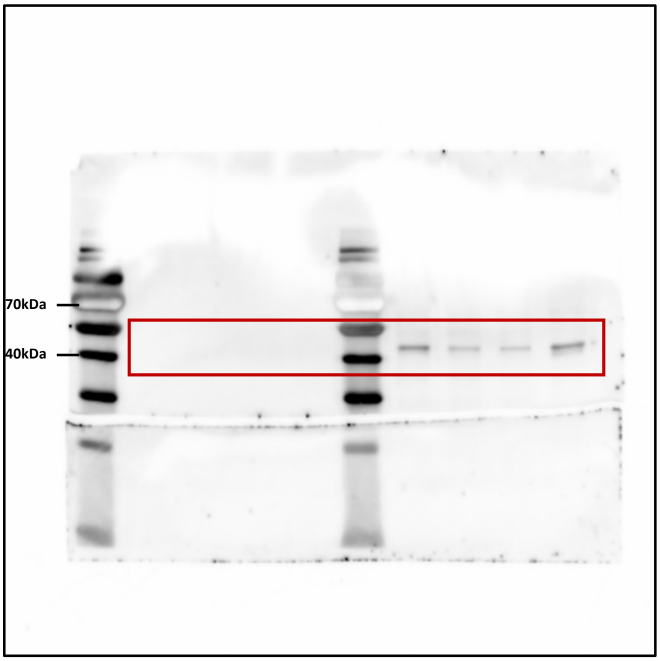

GRP94

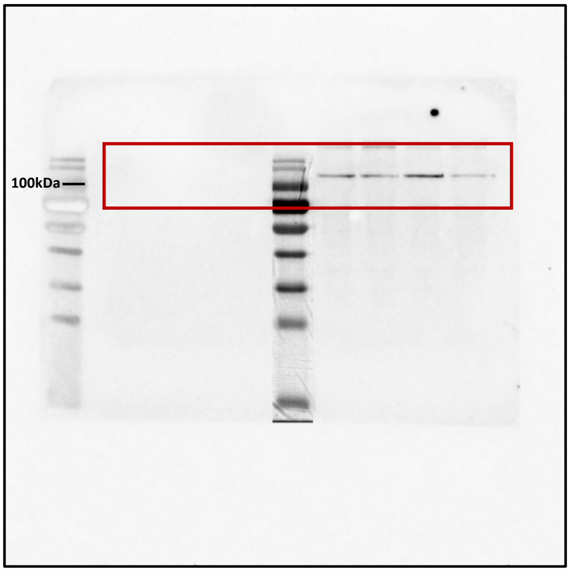

Supplementary Figure 11 (Related to Supplementary Figure 2). Characterization of EVs and cells. EVs and cellular protein markers evaluated by immunoblotting in EVs and cell lysate preparations from LNCaP cell lines.

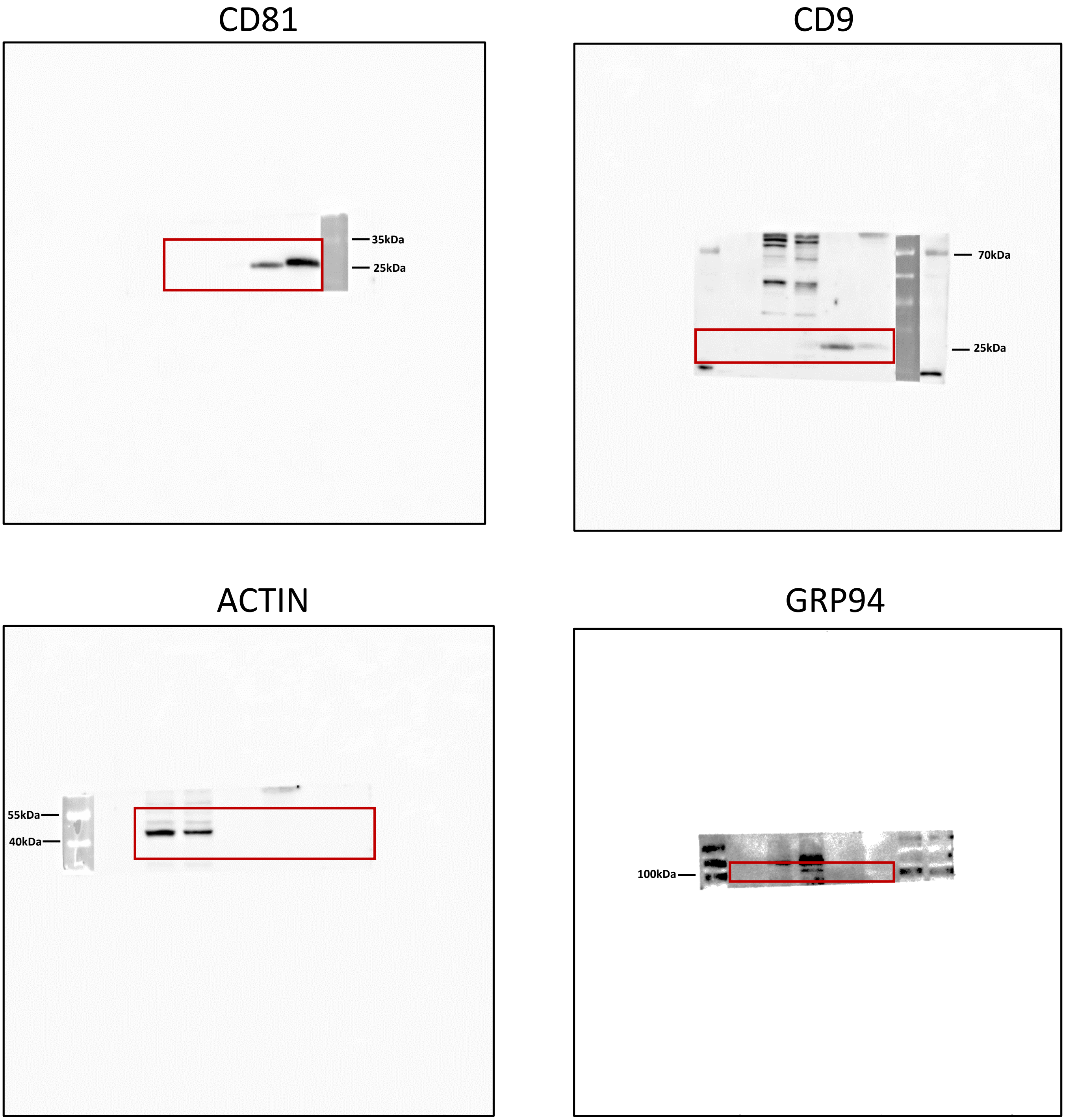

Supplementary Figure 12 (Related to Supplementary Figure 4). Characterization of EVs and cells. EVs and cellular protein markers evaluated by immunoblotting in EVs and cell lysate preparations from LNCaP-ABL and LNCaP parental cell lines.
