# Supplementary material for: Circulating extracellular vesicles release oncogenic miR-424 in experimental models and patients with aggressive prostate cancer
Source: Commun Biol. 2021 Jan 26;4:119. doi: 10.1038/s42003-020-01642-5 (PMC7838273; doi:10.1038/s42003-020-01642-5)
Supplement: Supplementary file 2 — Description of Supplementary Files [file 42003_2020_1642_MOESM2_ESM.pdf]

Description of Additional Supplementary Files

File name: Supplementary Data 1
Description: Clinical and pathological data of the patients included in the study.

File name: Supplementary Data 2
Description: List of genesUP/ DOWN_miR424 signature

File name: Supplementary Data 3
Description: Graph Source Data
