# Supplementary material for: Circulating extracellular vesicles release oncogenic miR-424 in experimental models and patients with aggressive prostate cancer
Source: Commun Biol. 2021 Jan 26;4:119. doi: 10.1038/s42003-020-01642-5 (PMC7838273; doi:10.1038/s42003-020-01642-5)
Supplement: Supplementary file 6 — Reporting Summary [file 42003_2020_1642_MOESM6_ESM.pdf]

nature research

Corresponding author(s): DBPR COMMSBIO-20-2079B

Last updated by author(s): 16/12/20

Reporting Summary

Nature Research wishes to improve the reproducibility of the work that we publish. This form provides structure for consistency and transparency in reporting. For further information on Nature Research policies, see our Editorial Policies and the Editorial Policy Checklist.

Statistics

For all statistical analyses, confirm that the following items are present in the figure legend, table legend, main text, or Methods section.

n/a	Confirmed	
☐	☒	The exact sample size (n) for each experimental group/condition, given as a discrete number and unit of measurement
☐	☒	A statement on whether measurements were taken from distinct samples or whether the same sample was measured repeatedly
☐	☒	The statistical test(s) used AND whether they are one- or two-sided<br>*Only common tests should be described solely by name; describe more complex techniques in the Methods section.*
☐	☒	A description of all covariates tested
☐	☒	A description of any assumptions or corrections, such as tests of normality and adjustment for multiple comparisons
☐	☒	A full description of the statistical parameters including central tendency (e.g. means) or other basic estimates (e.g. regression coefficient) AND variation (e.g. standard deviation) or associated estimates of uncertainty (e.g. confidence intervals)
☐	☒	For null hypothesis testing, the test statistic (e.g. F, t, r) with confidence intervals, effect sizes, degrees of freedom and P value noted<br>*Give P values as exact values whenever suitable.*
☒	☐	For Bayesian analysis, information on the choice of priors and Markov chain Monte Carlo settings
☐	☒	For hierarchical and complex designs, identification of the appropriate level for tests and full reporting of outcomes
☒	☐	Estimates of effect sizes (e.g. Cohen's d, Pearson's r), indicating how they were calculated

Our web collection on statistics for biologists contains articles on many of the points above.

Software and code

Policy information about availability of computer code

Data collection	Data were collected by using Microsoft Excel 2016 64-bit (Microsoft), NanoSight LM10 Nanoparticle Characterization System, MACSQuant Analyzer 10 flow cytometer (Miltenyi Biotec; Bergisch Gladbach, Germany), Jeol 1010 EX electron microscope (Jeol, Tokyo), MORADA digital camera system (Olympus, Tokyo), IMAGEJ, Zeiss Microscope with Canon EOS 450D camera, Living Image Software (IVIS Imaging Systems), ImageScope Slide Viewing APERIO Software 12.1.
Data analysis	Previously identified miR-424 associated gene signatures (including 1806 upregulated genes and 260 downregulated genes) 20, were investigated in a cohort of primary and castration resistant prostate cancer samples. RNA-Seq dataset (including 497 primary prostate cancers from TCGA (The Cancer Genome Atlas dataset (http://gdac.broadinstitute.org/) and 75 CRPCs (Fred Hutchinson Cancer Research Center) was used. Sequencing reads were aligned as previously described 29. Heat map were plotted performed in R environment (R Core Team. R: A Language and Environment for Statistical Computing. Vienna, Austria: R Foundation for Statistical Computing, 2013. http://www.R-project.org/. R package version 2.0.0). Principal Component Analysis was performed using the PCAtools (https://github.com/kevinblighe/PCAtools; Kevin Blighe and Aaron Lun (2020). PCAtools: Everything Principal Components Analysis. R package version 2.0.0. https://github.com/kevinblighe/PCAtools).

For manuscripts utilizing custom algorithms or software that are central to the research but not yet described in published literature, software must be made available to editors and reviewers. We strongly encourage code deposition in a community repository (e.g. GitHub). See the Nature Research guidelines for submitting code & software for further information.

Data

Policy information about availability of data

All manuscripts must include a data availability statement. This statement should provide the following information, where applicable:

- Accession codes, unique identifiers, or web links for publicly available datasets
- A list of figures that have associated raw data
- A description of any restrictions on data availability

Source data for the figures and extended data figures are provided. Source Data (gels) for all the WB reported in the paper are provided. All the other results can be available from the corresponding authors upon reasonable request. The datasets generated in the present study are available from the corresponding author upon reasonable request.

Field-specific reporting

Please select the one below that is the best fit for your research. If you are not sure, read the appropriate sections before making your selection.

[x] Life sciences [ ] Behavioural & social sciences [ ] Ecological, evolutionary & environmental sciences

For a reference copy of the document with all sections, see nature.com/documents/nr-reporting-summary-flat.pdf

Life sciences study design

All studies must disclose on these points even when the disclosure is negative.

Sample size	Sample sizes were estimated from other experiments obtained by similar experiments performed in former publications of the group.<br>Sample sizes for clinical data:<br>No statistical methods were used to predetermine sample sizes; sample sizes were selected based on the availability of specimens as specified below.<br>Blood was collected from patients with benign prostatic hyperplasia (BPH) (n=6), primary (n=25), metastatic castration sensitive (mCSPC) (n=16), and castration resistant prostate cancer (mCRPC) (n=17). Clinical samples were collected at the Portuguese Oncology Institute of Porto (Porto, Portugal), Groote Schuur, Eerste Rivier and New Somerset Hospitals (Cape Town, South Africa) and Oncology Institute of Southern Switzerland (Bellinzona, Switzerland). Samples were collected prior to any treatment at the time of diagnosis or during routine follow-up for metastatic patients. Metastatic disease was defined by radiologic progression, whereas CRPC was defined as previously described.<br><br>In vitro studies:<br>sample sizes were determined based on pilot studies results and on previous similar studies resulting statistically significant.<br><br>In vivo studies:<br>Animal experiments were conducted by using between 2 and 4 mice per group. This sample sizes in previously and similar studies, have given statistical significant results based on the variance of xenografts growth in control mice. For ethical reasons, the minimum number of animals necessary to achieve the scientific objectives was used.
Data exclusions	No data excluded
Replication	All experiments were performed at least two times and with sufficient biological and technical replicates with cells and animals per group in order to demonstrate statistical significance. Number of replicates in each experiment is indicated in the corresponding figure or legend.
Randomization	Animals were randomly subdivided for in vivo experiments and in exosomes injection studies, animals were randomized prior to systemic inoculation.
Blinding	All researcher were blinded during the counting of spheroids, colonies and xenografts measurements of the tumor size.

Reporting for specific materials, systems and methods

We require information from authors about some types of materials, experimental systems and methods used in many studies. Here, indicate whether each material, system or method listed is relevant to your study. If you are not sure if a list item applies to your research, read the appropriate section before selecting a response.

Materials & experimental systems

n/a	Involved in the study
☐	☒ Antibodies
☐	☒ Eukaryotic cell lines
☒	☐ Palaeontology and archaeology
☐	☒ Animals and other organisms
☐	☒ Human research participants
☐	☒ Clinical data
☒	☐ Dual use research of concern

Methods

n/a	Involved in the study
☒	☐ ChIP-seq
☐	☒ Flow cytometry
☒	☐ MRI-based neuroimaging

Antibodies

Antibodies used

CD9 (C-4, sc-13118, Santa Cruz Technology).
CD81 (EPR4244, ab109201, Abcam).
GRP94 (cat.#2104, Cell signalling technology).
β-actin (ab8226, Abcam).
Calnexin (AF18, cs-23954, Santa Cruz Technology).
GAPDH (cat.#0411, sc-47724, Santa Cruz Biotechnology).
STAT3 (124H6; Cell Signaling; catalog 9139).
p-STAT3 Tyr705 (D3A7; Cell Signaling; catalog 9145).
COP1 (ab56400, Abcam).
c-Jun (E254, ab32137).
Ki67 (Lab Vision Corp.; ready-to-use RT-9106-R7).

Validation

IHC was performed on 4μm formalin-fixed paraffin embedded (FFPE) tissue sections using an automated staining platform (Bond-RX, Leica Microsystems). Optimal antibody concentrations were determined with primary antibodies against STAT3 (#124H6; CELL SIGNALING, CATALOG 9139, dilution 1:100), p-STAT3 Tyr705 (#D3A7; CELL SIGNALING; CATALOG 9145 dilution 1:50), COP1 (#ab56400, Abcam, dilution 1:100), c-JUN (#E254, ab32137, Abcam, dilution 1:100), EZH2 (#D2C9, CELL SIGNALING; CATALOG 5246; dilution 1:200, and Ki67 (#RT-91066-R7, LAB VISION CORPORATION, ready-to-use. 3,3-diaminobenzidine tetrahydroxychloride (DAB) was used as chromogen and the slides were counterstained with hematoxylin. In each staining batch, positive and negative controls were incubated with and without primary antibody.

Eukaryotic cell lines

Policy information about cell lines

Cell line source(s)

LNCaP cells were obtained from ATCC and maintained in RPMI-1640 (Gibco) supplemented with 10% fetal bovine serum and 1% of penicillin. Immortalized human prostate epithelial cells RWPE-1 were obtained from ATCC and maintained in Keratinocyte serum-free growth medium (KSF; Gibco) with specific supplements. UGSM cells were obtained from ATCC and maintained in cells were maintained in DMEM/F12 + 10% FBS + 5% NuSerum IV, 1x glutamine, 1x penicillin/streptomycin solution, 10−8 M (0.01μM) final concentration of Dihydrotestosterone, 25ug ml−1 insulin. LNCaP/RWPE-1 stably expressing miR-424 (LNCaP/RWPE-1-424) and empty vector (EV) as control (LNCaP/RWPE-1-EV) were established as previously described (Dallavalle et al, 2016). LNCAPabl were grown in Charcoal stripped serum (CSS) as previously described (Groner, A.C., et al 2016).

Authentication

All cell lins used were authenticated by the provider.

Mycoplasma contamination

All cell lines were tested for mycoplasma contamination periodically, via the MycoAlert Mycoplamsa Testing kit (Lonza). Results were always negative for mycoplasma contamination.

Commonly misidentified lines
(See ICLAC register)

No commonly misidentified cell lines were used in present study.

Animals and other organisms

Policy information about studies involving animals; ARRIVE guidelines recommended for reporting animal research

Laboratory animals

Male of NOD.Cg-PrkdcSCID Il2rgtm1Wjl/SzJ (NSG-KO) mice (4–6 weeks old, Jackson Laboratories) were used for xenografts establishment. The PbCre4; Pten flox/+ R26LSL;ERG mouse was generously provided by Dr. Charles L. Sawyers and maintained as previously described (Shinde, D., et al 2019).

Wild animals

No wild animals were used.

Field-collected samples

No field-collected samples were used.

Ethics oversight

All mouse studies were approved by the MSKCC Institutional Animal Care and Use Committee under protocol 06-07-012.

Note that full information on the approval of the study protocol must also be provided in the manuscript.

Human research participants

Policy information about studies involving human research participants

Population characteristics	Samples were collected prior to any treatment at the time of diagnosis or during routine follow-up for metastatic patients. Metastatic disease was defined by radiologic progression, whereas CRPC was defined as previously described (22). Plasma of patients (n=64) with benign prostatic hyperplasia (BPH) (n=6), primary (n=25), metastatic castration sensitive (mCSPC) (n=16) and hormone-refractory (mCRPC) (n=17) prostate cancers was collected to isolate exosomes. Average age at diagnosis: 70.48 years. Metastatic sites: Bone, Lymph node. Gleason: N=29 with 8-10, N=24 with 7, N=2 <6, N=9 NS.
Recruitment	Clinical and pathological data (Supplementary table 1) were collected in an anonymized database.
Ethics oversight	Ethical approval for the study was obtained by the ethical committees of the individual collaborating Institutions: Comissão de Ética para a Saúde (CES-IPOFG-EPE 205/2013, IPO Porto), Human Research Ethics Committee (HREC454/2012, University of Cape Town) and Ethical Committee of Canton Ticino (CE TI 3269 11 Project-ID 2017-01631, IOSI).

Note that full information on the approval of the study protocol must also be provided in the manuscript.

Clinical data

Policy information about clinical studies

All manuscripts should comply with the ICMJEguidelines for publication of clinical research and a completedCONSORT checklist must be included with all submissions.

Clinical trial registration	*Provide the trial registration number from ClinicalTrials.gov or an equivalent agency.*
Study protocol	*Note where the full trial protocol can be accessed OR if not available, explain why.*
Data collection	*Describe the settings and locales of data collection, noting the time periods of recruitment and data collection.*
Outcomes	*Describe how you pre-defined primary and secondary outcome measures and how you assessed these measures.*

Flow Cytometry

Plots

Confirm that:

- [ ] The axis labels state the marker and fluorochrome used (e.g. CD4-FITC).
- [ ] The axis scales are clearly visible. Include numbers along axes only for bottom left plot of group (a 'group' is an analysis of identical markers).
- [ ] All plots are contour plots with outliers or pseudocolor plots.
- [ ] A numerical value for number of cells or percentage (with statistics) is provided.

Methodology

Sample preparation	*Describe the sample preparation, detailing the biological source of the cells and any tissue processing steps used.*
Instrument	*Identify the instrument used for data collection, specifying make and model number.*
Software	*Describe the software used to collect and analyze the flow cytometry data. For custom code that has been deposited into a community repository, provide accession details.*
Cell population abundance	*Describe the abundance of the relevant cell populations within post-sort fractions, providing details on the purity of the samples and how it was determined.*
Gating strategy	*Describe the gating strategy used for all relevant experiments, specifying the preliminary FSC/SSC gates of the starting cell population, indicating where boundaries between "positive" and "negative" staining cell populations are defined.*

- [ ] Tick this box to confirm that a figure exemplifying the gating strategy is provided in the Supplementary Information.
